# Characterising functional redundancy in microbiome communities via relative entropy

**DOI:** 10.1016/j.csbj.2025.03.012

**Published:** 2025-03-12

**Authors:** Daniel Fässler, Almut Heinken, Johannes Hertel

**Affiliations:** a Department of Psychiatry and Psychotherapy, University Medicine Greifswald, Greifswald, Germany; b UMRS Inserm 1256 nGERE (Nutrition-Genetics-Environmental Risks), Vandœuvre-les-Nancy, France; c German Centre for Cardiovascular Research (DZHK), Partner Site Greifswald, Greifswald, Germany

**Keywords:** Functional redundancy, Microbiome function, Microbiome diversity, Relative entropy, Constraint-based community modelling, Information theory

## Abstract

Functional redundancy has been hypothesised to be at the core of the well-evidenced relation between high ecological microbiome diversity and human health. Here, we conceptualise and operationalise functional redundancy on a single-trait level for functionally annotated microbial communities, utilising an information-theoretic approach based on relative entropy that also allows for the quantification of functional interdependency across species. Via constraint-based microbiome community modelling of a public faecal metagenomic dataset, we demonstrate that the strength of the relation between species diversity and functional redundancy is dependent on specific attributes of the function under consideration such as the rarity and the occurring functional interdependencies. Moreover, by integrating faecal metabolome data, we highlight that measures of functional redundancy have correlates in the host’s metabolome. We further demonstrate that microbiomes sampled from colorectal cancer patients display higher levels of species-species functional interdependencies than those of healthy controls. By analysing microbiome community models from an inflammatory bowel disease (IBD) study, we show that although species diversity decreased in IBD subjects, functional redundancy increased for certain metabolites, notably hydrogen sulphide. This finding highlights their potential to provide valuable insights beyond species diversity. Here, we formalise the concept of functional redundancy in microbial communities and demonstrate its usefulness in real microbiome data, providing a foundation for a deeper understanding of how microbiome diversity shapes the functional capacities of a microbiome.

## Graphical Abstract

**Figure:**
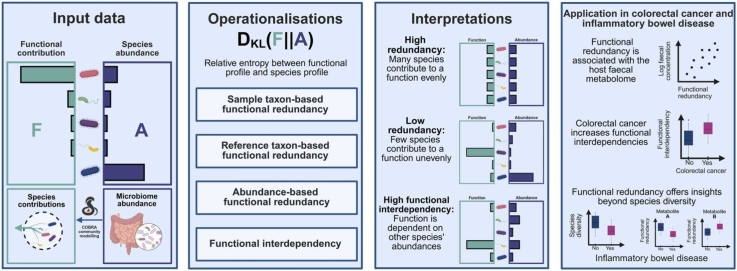

## 1 Introduction

To understand the multitude of functions of the microbiome, ecological diversity represents a key concept. Ecological diversity encompasses various dimensions of diversity and is often used synonymously with species diversity in microbiome research, which describes the richness of species within a community and the evenness of their distribution [4], [5]. Multiple measures, such as Shannon entropy or Simpson’s diversity, have become essential in microbiome research [6], [7], [8], [9], [10]. However, summarising species diversity with a single measure is problematic [11], and the question remains as to how informative diversity measures are and what they capture [12]. Measures of species diversity rely solely on taxonomic information and compositional variation, and do not directly reflect functional diversity or functional redundancy of a microbial community [13], [14], [15]. Hence, two microbial communities may diverge strongly in their composition and their species diversity but be largely equivalent in their metabolic functions [16], [17], [18]. A compositionally highly diverse community can have low functional redundancy, just as a microbial community with lower species diversity can be rich in functional redundancy. Nevertheless, a range of research has asserted that high species diversity leads to more stable host-microbiome interactions through functional redundancy [19], [20], aiming to explain the wealth of evidence for a range of human diseases being associated with reduced microbiome species diversity [21], [22], [23], [24].

Functional diversity and redundancy have been investigated previously by incorporating pairwise functional dissimilarities among co-existing species based on multiple traits [25], [26], [27], [28], [29]. However, selecting relevant functional traits while excluding non-informative ones is challenging, as this directly affects the accuracy and ecological relevance of functional diversity measures [30]. Moreover, in microbiome research, we are often interested in individual functions that are core to certain physiological aspects of host-microbe interactions [31], [32], [33].

Here, we target functional redundancy at the single-trait level by introducing information-theoretic operationalisations that can be realised via detailed functional and quantitative annotations as delivered by constraint-based microbiome community modelling based on genome-scale reconstructions [34], [35], [36].

Genome-scale metabolic models of microorganisms [37], [38], [39], [40] based on constraint-based reconstruction and analysis [34], [36], [41] (COBRA) provide a powerful resource to capture metabolic functions of microbiomes and can be applied to generate microbiome communities by integrating personalised metagenomic data. COBRA-based community modelling delivers a detailed quantitative annotation of metabolic functions across a wide range of biochemical pathways and can be utilised to quantify the maximum metabolite secretion potential of the community as a whole and of each individual microbe [42], [43]. Corresponding predictions in the form of maximal net secretion fluxes have been shown to hold predictive power through the integration of metabolome data in multiple works [37], [44], [45], [3].

Applying our methodology to two public datasets, we will demonstrate that the derived operationalisation provides meaningful insights not only into the theoretical and empirical relations between species diversity and functional redundancy but also into host-microbiome interactions in colorectal cancer (CRC) and inflammatory bowel disease (IBD).

## 2 Theoretical concepts

To motivate the chosen operationalisation of functional redundancy, we first define functional redundancy for a given function of a microbial community in biological terms. Intuitively, if many species or organisms in a community are able to perform a certain function, such that the loss of some of these species or organisms does not lead to the loss of the function, we would call the community functionally redundant with respect to that function. Therefore, we define functional redundancy as follows:

Definition 1 Functional redundancy of a specific function in microbial communities

Functional redundancy of a certain function describes the potential of a microbial community to retain that function under the loss of microbial biomass.

Thus, we understand functional redundancy as the projected capacity of a microbial community to perform *a certain function* (e.g., butyrate production) if the community would experience the depletion or the entire loss of microbial species, in contrast to previous conceptualisations that understand functional redundancy as a multi-trait attribute [28], [46], [47].

An important aspect of our definition is that it can be operationalised in different ways. First, we can characterise functional redundancy on the level of taxonomic units, meaning, for example, that multiple species capable of performing the same function are present in a given community (taxon-based functional redundancy). Second, we can characterise functional redundancy by the abundance of organisms that can perform a function in a given community (abundance-based functional redundancy). Both characterisations have their merits. Which one is more appropriate depends on the biological phenomenon under investigation and the available data. For example, consider a community where only a single but highly abundant species can perform a certain function. In terms of taxon-based functional redundancy, functional redundancy would be minimal, as a single species is responsible for upholding the function, and thus the loss of this species would result in the entire loss of the function. In terms of abundance-based functional redundancy, in contrast, the functional redundancy may be considerable, as one may lose a lot of biomass without losing the function. Depending on the type of biomass loss we expect to be relevant for a certain community, we may choose one operationalisation over the other. Thus, we provide a formal framework for both operationalisations.

### 2.1 Formalisation

We formalise functional redundancy for a microbial community containing n-species, denoted by $S=\{S_{1},{\ldots ,S}_{n}\}$, with relative abundances $a=(a_{1},{\ldots ,a}_{n})\in {\left(0,1\right]}^{n}$. For each species in the community, we consider a quantitative output of a specific function F, denoted as $f=\left(f_{1},\ldots ,f_{n}\right)\in {\left[0,\gamma \right]}^{n}$, where γ is the maximum of the function of interest. Let $\tilde{f}=\left({\tilde{f}}_{1},\ldots ,{\tilde{f}}_{n}\right)$, where ${\tilde{f}}_{i}=\frac{f_{i}}{f_{\mathrm{total}}}\in \left[0,1\right]$, be the vector of shares of each species $S_{i}$ for the function F relative to the total community output $f_{\mathrm{total}}=\overset{n}{\underset{i=1}{\sum }}f_{i}>0$. Furthermore, let $S_{\mathrm{ref}}$ be a reference set containing all m-species that can perform the function in theory. We analogously define $f_{\mathrm{ref}}$ and correspondingly ${\tilde{f}}_{\mathrm{ref}}$ as the quantitative output and corresponding shares based on the reference $S_{\mathrm{ref}}$. Applied to a concrete microbial community, f and $f_{\mathrm{ref}}$ will only differ in the number of zeros, as each species in the microbial community capable of performing the function must be present in the reference set and vice versa.

#### 2.1.1 Taxon-based functional redundancy

We search for measures that reach their maximum, when each species of the system performs the same quantitative amount of the function of interest, and their minimum if and only if a single species can perform the function. We search for a mapping $R:(f_{1},\ldots ,f_{n})\to \mathbb{R}$ that is maximised, when $0<f_{1}\;=\;f_{2}=\;\ldots \;=\;f_{n}$ and minimised, when $f_{j}>0$, for one $j\in \left\{1,..,n\right\}$ and $f_{i}=0$, for all i≠j. Here, we propose to utilise an information-theoretic operationalisation that satisfies the above mentioned criteria and uses the functional shares $\tilde{f}$.

The taxon-based functional redundancy in respect to the function F is then given by:

(1) $$R_{\mathrm{Taxon}}={-D}_{\mathrm{KL}}\left(\overset{\sim }{f}\;\|U_{n}\right)={H\left(\overset{\sim }{f}\right)-H}_{\max}\left(\overset{\sim }{f}\right)=-\underset{i:{\overset{\sim }{f}}_{i}>0}{\sum }{\overset{\sim }{f}}_{i}\;\log{(\overset{\sim }{f}}_{i})-\log\left(n\right)$$

Here, ${-D}_{KL}\left(\overset{∼}{f}||U_{n}\right)$ denotes the relative entropy or Kullback-Leibler divergence between $\tilde{f}$ and a discrete uniform distribution $U_{n}$, $H_{\max}\left(\tilde{f}\right)$ the maximal possible entropy of $\tilde{f}$, $H\left(\tilde{f}\right)$ the actual entropy of $\tilde{f}$, and n the number of species in the microbial community (Supplementary Material). Note that we use the negative relative entropy to ensure that a higher functional redundancy measure corresponds to higher functional redundancy. The functional redundancy measure $R_{\mathrm{Taxon}}$ is negative, or zero in case of maximum functional redundancy. Hence, we define functional redundancy analogously to absolute redundancy in information theory, where it is defined as the difference between maximum entropy and actual entropy [48].

The above-defined measure of taxon-based functional redundancy is difficult to compare across communities of different species richness, as the measure depends heavily on the species richness of the community under consideration. This problem can be circumvented by utilising ${\tilde{f}}_{\mathrm{ref}}$ as defined above:

(2) $$R_{\mathrm{Taxon}}={-D}_{\mathrm{KL}}\left({\overset{∼}{f}}_{\mathrm{ref}}||U_{m}\right)=-\underset{i:{\overset{∼}{f}}_{i}>0}{\sum }{\overset{∼}{f}}_{i}\log{(\overset{∼}{f}}_{i})-\log(m),$$

where m is the number of species in the reference, fixed for each function, across all microbiome communities. We refer to Eqn 1 as *sample* taxon-based functional redundancy and to Eqn 2 as *reference* taxon-based functional redundancy.

#### 2.1.2 Abundance-based functional redundancy

Abundance-based functional redundancy is characterised by the distribution of individual organisms capable of performing the function within a given microbial community. Maximum functional redundancy would be attained if each organism would contribute equally to the total community output, while minimal functional redundancy would be attained if only one organism would be responsible for the entire community output. The abundance-based functional redundancy is then given by:

(3) $${R_{\mathrm{Abundance}}=-D}_{\mathrm{KL}}\left(\overset{∼}{f}||a\right)=-\underset{i:{\overset{˜}{f}}_{i}>0}{\sum }{\overset{∼}{f}}_{i}\log\left(\frac{{\overset{∼}{f}}_{i}}{a_{i}}\right).$$

Thus, Eqn 3 represents the relative entropy between the relative shares of species in terms of total community output and the abundance vector. This measure is maximal if each $f_{i}$ is a linear function of its corresponding abundance $a_{i}$, i.e., it must hold that $f_{i}=ba_{i}+d$, where b and d are constants across all i∈{1,…,n}.

#### 2.1.3 The functional interdependency index

Going one step further, we can ask how $D_{\mathrm{KL}}\left(\overset{∼}{f}||a\right)$ relates to the total abundance of k species capable of performing a certain function. Indeed, one can show that $D_{\mathrm{KL}}\left(\tilde{f}∥a)\right)$ is a direct function of $\sum_{i=1}^{k}a_{i}$, if all $f_{i}$ are *only dependent* on $a_{i}$ (Supplementary Methods, Attribute 1). This scenario represents a case of missing species-species interdependencies with respect to the shares $\tilde{f}$, conceptualising interdependency as the dependency of a function on the abundances of more than one species. Thus, this formalisation enables a quantification of the intensity of functional interdependencies in a microbial community. To this end, we define the functional interdependency index I by considering the sub-system of all species with shares greater zero: ${\left({\overset{∼}{f}}_{j}\right)}_{j\in J}=({{\overset{∼}{f}}_{j}}_{1},\ldots ,{{\overset{∼}{f}}_{j}}_{k})$, where $J=\{j_{1},\ldots ,j_{k}\}=\{i:{\overset{˜}{f}}_{i}>0\}$. The functional interdependency index is defined as the relative entropy between the shares vector of functions greater zero and its associated vector of restricted abundances. Let $\tilde{f_{J}}={\left({\tilde{f}}_{j}\right)}_{j\in J}$ and ${\tilde{a}}_{J}={\left({\tilde{a}}_{j}\right)}_{j\in J}$, where ${\tilde{a}}_{j}=\frac{a_{j}}{\sum_{j\in J}a_{j}}$. The interdependency index I is calculated as:

(4) $${I=D}_{\mathrm{KL}}\left(\overset{∼}{f_{J}}||{\overset{∼}{a}}_{J}\right)=\underset{j\in J}{\sum }{\overset{∼}{f}}_{j}\log\left(\frac{{\overset{∼}{f}}_{j}}{{\overset{∼}{a}}_{j}}\right).$$

The functional interdependency index delivers a measure of the intensity of interdependencies that may also occur between more than two microbes, quantifying an aspect which has been hard to measure so far.

#### 2.1.4 The global functional interdependency index

On functional interdependency indices for multiple functions of a sample, we define a global index of functional interdependency to measure functional interdependency at a community-level across all functions. Let $I_{1},\ldots ,I_{n}$ represent multiple functional interdependency indices across n functions of the same sample. We define the global functional interdependency index as the median of $\left\{I_{1},\ldots ,I_{n}\right\}$:

(5) $$I_{g}=\mathrm{med}\left(\left\{I_{1},\ldots ,I_{n}\right\}\right),$$

where the median is used for robustness against outliers and potentially skewed distributions. However, other metrics of central tendency could also be employed.

**Fig. 1:**
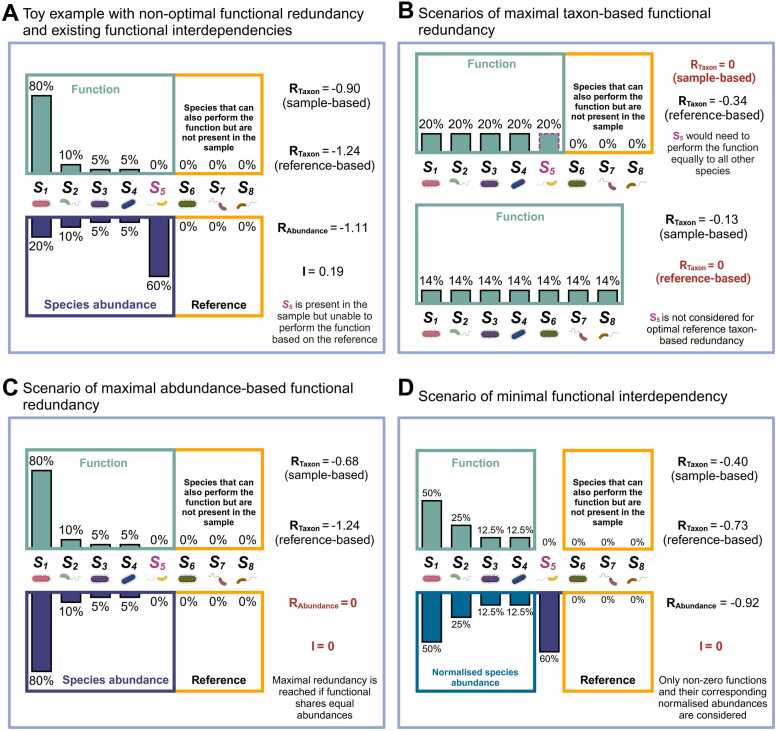
Overview of a toy example, illustrating scenarios of maximal functional redundancy and minimal functional interdependency. A, Introduction of the toy example which consists of five species in the sample, four species that can perform the function in the sample, and three additional species in the reference that can perform the function but are not present in the sample (non-optimal functional redundancy). B, All species in the sample share an equal amount of the function (maximal sample taxon-based functional redundancy, upper part), and all species capable of performing the function are present in the sample and contribute equally to the total function share (maximal reference taxon-based redundancy, lower part). C, All organisms in the sample share the same function equally. The amount of function shared corresponds to the abundance of each species (maximal abundance-based functional redundancy). D, The abundance of each species present is normalised based on the total abundance of species capable of performing the function. With minimal functional interdependency, each species in the sample shares the function in accordance with the normalised species abundance, and the species’ abundance completely explains the species’ functional share (minimal functional interdependency).

Fig. 1 provides a graphical overview of the introduced concepts and operationalisations using a toy example. A more detailed discussion of this toy example, including further illustrations and example calculations, can be found in the Supplementary Material along with fundamental mathematical attributes of the proposed operationalisations.

## 3 Materials & methods

### 3.1 Study samples

#### 3.1.1 Colorectal cancer study

For an empirical application of our approach, we utilised a Japanese colorectal cancer (CRC) cohort dataset from Yachida et al. [1]. This study provided publicly available shotgun faecal metagenomic sequencing data for n = 616 individuals (365 CRC cases and 251 healthy controls), as well as mass spectrometric metabolome data, including quantifications for 450 metabolites in stool. Sequencing reads and taxonomic assignments were processed using the MetaPhlAn2 [49] pipeline. The dataset included several metadata of which age, sex, and BMI were included in our analysis. Details on metagenomic and metabolomics measurements, along with the raw metabolome and microbiome abundance table can be found in the supplementary material of the stem publication.

#### 3.1.2 Pediatric Crohn’s disease cohort

We utilised data from a prospective cohort of pediatric Crohn’s disease patients reported by Lewis et al. [2]. Specifically, we utilised individual maximum secretion fluxes of the species in each of 108 microbiome community models previously generated in Heinken et al. [3]. The community models correspond to 20 samples characterized as inflammatory bowel disease (IBD) microbiomes with dysbiosis, 63 non-dysbiotic IBD microbiomes, and 25 control samples, with no additional metadata provided.

### 3.2 Generation of sample-specific COBRA community models for the CRC study

Functional annotations were derived through COBRA community modelling. For the CRC study, the relative abundances obtained from Yachida et al. [1] were mapped onto the reference set of 818 microbial metabolic reconstructions (AGORA [38]). A more detailed explanation of generating COBRA community models is noted in Hertel et al. [44]. Briefly, the created (pan-)models of AGORA reconstructions of microbes present in a sample were connected through a shared lumen compartment to create a microbiome community model of each sample. Each community model was parameterised using the biomass reaction of each genome-scale reconstruction present, weighted by their relative abundances, to derive a community biomass reaction. Diet constraints were applied by constraining the lower bound of the corresponding sink reaction of the diet compartment corresponding to an average Japanese Diet as described previously [44]. Each model was built in MATLAB (R2018b, MathWorks, Inc.) using the COBRA toolbox [41] and the Microbiome Modeling Toolbox [42], [50]. Personalised models for the 108 IBD samples were also generated using AGORA reconstructions, following the same methodology applied to the CRC study [3].

### 3.3 Calculation of individual maximum secretion fluxes of COBRA community models

The maximum secretion fluxes for the CRC study were computed for each metabolite in every COBRA community model where at least one exchange reaction into the lumen compartment was available via the predictMicrobeContributions function of the COBRA toolbox. The function employs Flux Variability Analysis (FVA) to determine the maximum and minimum fluxes of internal exchange reactions into the lumen compartment, with the community biomass serving as the initial objective function, maximised and subsequently held constant. The function provided individual maximal secretion fluxes for 91 metabolites across all communities and microbes in the CRC study. The computed individual secretion fluxes for the finally analysed metabolites can be found in the Table S1. We inverted the sign of the output values, such that a positive value of each flux represents secretion, and a negative value represents uptake, following this convention throughout the text. All simulations were performed in MATLAB (Mathworks, Inc.) version R2021a with IBM CPLEX (IBM) as the linear programming solver. A formal operationalisation of functional redundancy measures based on the computed individual secretion fluxes can be found in the Supplementary Material.

### 3.4 Calculation of functional redundancies in the IBD and CRC study

All functional redundancy and functional interdependency measures, simulations, and statistical analyses were conducted using R version 4.4.2. We have created an R package, *FunRed,* to calculate functional redundancy and interdependency measures, which includes instructions and descriptions of each index, demonstrated using the toy example from the manuscript (with corresponding input/output). The package is available at https://github.com/SysPsyHertel/FunRed. For calculating the Kullback-Leibler divergence for our redundancy measures, we used the KL function of the *philentropy* [51] package with default parameters, except for setting unit = "log" to compute divergence using the natural logarithm. We normalised the abundances for each sample prior to computing functional redundancy and functional interdependency to ensure that the shares summed up to 1. For computing Shannon entropy and Simpson’s diversity we utilised the *vegan* [52] package. We calculated species evenness by dividing Shannon entropy by the logarithm of species richness. All calculations and measures reliant on abundances were calculated exclusively from the abundance of the species mapped onto AGORA.

We excluded samples with fewer than 25 species in their respective community models, resulting in 612 analysed community models for the CRC study (out of 616) and 102 community models for the IBD study (out of 108). The COBRA community models were constructed at the species level for the CRC study and at the strain level for the IBD study. For each community model, we set measures of functional redundancy and functional interdependency as missing if in a simulation of a sample-metabolite pair, at least one microbe had to take up the metabolite to optimise the community biomass reaction, or if there were no microbes in the community capable of secreting the metabolite. Subsequently, for the analyses of metabolic functional redundancy measures, we exclusively considered metabolites with over 80 % non-missing values of functional redundancy across the community models. This criterion led to the inclusion of 45 metabolites for the CRC study and 161 metabolites for the IBD study.

### 3.5 Statistical analysis

For descriptive purposes, metric variables were characterised by means and standard deviations and categorical variables by proportions. For all analyses, we used the natural logarithm for any log transformation. P-values are reported as two-sided. For multiple testing correction, we utilised the Bonferroni correction. The significance level was set to α=0.05 or $\frac{0.05}{\mathrm{\#tests}}$ when adjusting with the Bonferroni method. Bonferroni-adjusted p-values in the supplement were reported using the p.adjust function of R. Linear regressions were performed using the standard lm function in R. For visualisations of regression results, regression fits are displayed with 95 % confidence bands.

#### 3.5.1 Statistical analysis of the CRC study

To test the relation between the three functional redundancy measures with each other, we performed regressions across all three possible combinations without any adjustments. Furthermore, we fitted linear regressions with the intercepts of the regressions between reference and sample taxon-based functional redundancy measures as the dependent variable and the rarity of a function (e.g., the number of producing species) as the sole predictor. To explore the statistical relations between functional redundancy and species diversity, we used linear regression models, treating Shannon entropy as response variable and the respective functional redundancy measure as predictor of interest, without adjusting for additional covariates. For testing on associations between functional redundancy measures and faecal concentrations, we log-transformed the faecal concentrations. Of the 45 metabolites for which we calculated functional redundancy measures, 21 metabolites had non-missing faecal measurements in at least 50 % of cases after log transformations and were included in the analysis. We used linear regression models with the log-scaled faecal concentrations as response variable, the corresponding functional redundancy measure as predictor of interest and adjusted for possible confounders: Age, BMI, sex, and health status (CRC: yes/no). Permutation tests were performed by randomly permuting the corresponding redundancy measure 10,000 times, refitting the linear model on each permuted dataset, and calculating the p-value by comparing the observed coefficient’s p-value to the distribution of p-values obtained from the permuted datasets. For the analyses of CRC health status associations with functional redundancy and interdependency, each functional redundancy measure and the log-transformed interdependency index were treated as response variables in a separate linear regression model. Health status was chosen as the predictor of interest for each model while adjusting for age, BMI and sex.

#### 3.5.2 Statistical analysis of the IBD study

We performed an analysis of variance (ANOVA) using the standard *aov* function in R to assess differences among the three stratification groups. Planned contrasts were applied to compare the average between IBD patients and healthy controls, with the contrast defined using weights (-1, 0.5, 0.5) representing (Healthy, IBD non-dysbiotic, IBD dysbiotic). The contrasts and analyses were implemented using the *emmeans* [53] package. Linear regressions were conducted, treating the corresponding redundancy-measure as the response variable and the disease group as independent variable while adjusting for Shannon entropy. Wald tests were performed for determining the significance of the three-valued categorical variable using the *car* package in R.

### 3.6 Comparing community-level functional redundancy with trait-based functional redundancy

For computing community-level functional redundancy based on Rao’s quadratic entropy, we used the rao.diversity function of the *SYNCSA* [54] R package, which, by default, calculates the dissimilarity matrix using the square root of the one-complement of Gower’s similarity. Functional redundancy in this sense can be defined as the portion of a community’s alpha taxonomic diversity not explained by its alpha functional diversity. A commonly used approach is to quantify the difference between Simpson’s diversity and functional diversity (represented by Rao’s quadratic entropy): FR=D−Q, where D represents Simpson’s diversity ($D=1-{\sum_{i=1}^{n}a_{i}}^{2}$), and Q represents Rao’s quadratic entropy ($Q=\sum_{i=1}^{n}\sum_{j=1}^{n}d_{\mathrm{ij}}a_{i}a_{j})$, for a community containing n-species. Thus:

(6) $$\mathrm{FR}=1-{\sum_{i=1}^{n}a_{i}}^{2}-\sum_{i=1}^{n}\sum_{j=1}^{n}d_{\mathrm{ij}}a_{i}a_{j},$$

with $d_{\mathrm{ij}}$, i,j∈{1,…,n} being dissimilarity weights between species $S_{i}$ and $S_{j}$, i,j∈{1,…,n} ranging from 0 (no dissimilarity) to 1 (complete dissimilarity), with $d_{\mathrm{ii}}=0$ for all i∈{1,…,n}. When species traits are maximally dissimilar ($d_{\mathrm{ij}}=1$, for all i≠j), Q is maximal and equals Simpson’s diversity, D. The value of FR is always scaled from 0 to 1, where 0 indicates minimal redundancy and 1 represents maximal redundancy.

### 3.7 Simulation for characterising the effect of unclassified species with unknown contributions

For simulating the effect of unclassified species and for comparison with community-level functional redundancy measures, we utilised synthetic communities sampled from Dirichlet distributions using the rdirichlet function of the *MCMCpack* [55] R package. For simulating unknown species, we first simulated 1000 iterations of functional contributions of 100 species with $\tilde{f}=\left({\tilde{f}}_{1},\ldots ,{\tilde{f}}_{100}\right)$ and abundances $a=\left(a_{1},\ldots ,a_{100}\right)$. The parameters $\alpha_{1},\ldots ,\alpha_{100}$ of the Dirichlet distribution were set randomly with a probability of 50 % either to ${\alpha =\alpha }_{1}=\ldots =\alpha_{100}=1$ (indicating an uneven distribution) or to ${\alpha =\alpha }_{1}=\ldots =\alpha_{100}=5$ (indicating a more even distribution). Next, we randomly reclassified 10 species and their contributions as “unknown”. Afterward, we recalculated the measures of functional redundancy on the normalised and shortened vectors (having now only 90 entries) and repeated this process iteratively until a total of 80 species had been reclassified as unknown.

## 4 Results

We structure the results in three parts. First, we explore the relation of the introduced functional redundancy measures to previously utilised community-level functional redundancy operationalisations [28], [46], [47], [56], [57]. Second, we provide simulations to investigate the effects of unclassified species with unknown functions on the information-theory based functional redundancy measures. Finally, we present the results of the analysis of microbiome functional redundancies in CRC and IBD.

### 4.1 Trait-based functional redundancy and community-level functional redundancy

First we demonstrate that the introduced single-trait functional redundancy measures are neither mathematically nor conceptually a special case of equation of the multi-trait community-level functional redundancy measures as formalised in Eq. (6). To this end, we calculate the dissimilarity weights $d_{\mathrm{ij}}$ in Eq. (6) based on one single function, and compare both approaches in an extreme case of one keystone species being solely responsible for a certain function among a reference of 100 species.

**Fig. 2:**
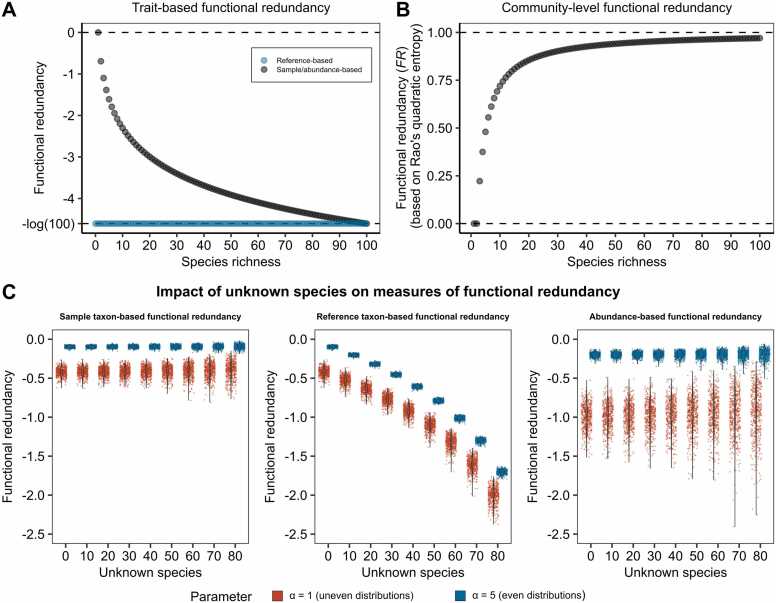
Functional redundancy illustrated with a single species performing a function while increasing species richness with evenly distributed abundances for both, trait-based functional redundancy and community-level functional redundancy and the effect of unknown species on functional redundancy measures. A, Trait-based functional redundancy as captured by our derived functional redundancy measures. The dashed line represents minimal functional redundancy (−log(100)) and maximal functional redundancy (0). The reference-based functional redundancy measure remains minimal and independent of species richness (blue), while sample- and abundance-based redundancy decrease from maximal functional redundancy (one species in the community) to minimal functional redundancy (100 species in the community). B, Community-level functional redundancy based on Rao’s quadratic entropy. Functional redundancy (FR) is minimal with a single species and increases monotonically with species richness, converging to maximal functional redundancy of FR (1). C, Simulations for the three redundancy measures with varying the alpha parameter, where α=5 representing more even distributions and α=1 more uneven ones.

We now calculated the functional redundancies for a series of increasing species richness, from 1 to 100, such that ${\tilde{f}}_{\#1}=\left(1\right)=a_{\#1},$ ${\tilde{f}}_{\#2}=\left(1,0\right)$ and $a_{\#2}=\left(\frac{1}{2},\frac{1}{2}\right)$, continuing until ${\tilde{f}}_{\#100}=\left(1,0,\ldots ,0\right)$ and $a_{\#100}=\left(\frac{1}{100},\ldots ,\frac{1}{100}\right)$. In this scenario, reference taxon-based functionality is always minimal and therefore −log(100) (Supplementary Material, Attribute 2), since regardless of the species diversity and species richness, only one species can contribute to the function (Fig. 2A). If this species would be lost, the community would lose the entire function, which refers to a case of minimal functional redundancy with respect to the investigated trait. In contrast, FR as calculated by Eq. (6) increases strongly with the size of the community (Fig. 2B), and thus does not reflect this scenario of minimal functional redundancy.

The sample taxon-based and abundance-based measures, being equivalent in this specific scenario (Supplementary Material, Attribute 3), behave very differently to FR as well (Fig. 2A). With increasing species richness, sample taxon-based and abundance-based functional redundancy will decrease, as the trait is dependent on a decreasing share of the total abundance and/or number of species, while FR will increase. In conclusion, multi-trait measures such as Eq. (6) do not capture functional redundancy in the sense of Definition 1 in contrast to the operationalisation introduced in this manuscript.

### 4.2 Impact of unknown species on measures of functional redundancy

An important problem in microbiome analysis is the presence of unclassified species with unknown functional contributions. Attribute 4 (Supplementary Material) shows that an increasing number of unclassified species will introduce an overestimation of the true functional redundancy if those species do not contribute to the function of interest. Thus, with rare functions, where unclassified species will be unlikely to exhibit the function, higher shares of unclassified species will lead to overestimation of the true functional redundancy. However, for other situations, the bias will be highly dependent on the distributional aspects of f and ***a***, making it challenging to derive insights via closed-form solutions.

To nevertheless derive insights on the effect of unclassified species in a more general setting, we performed 1000 simulations of communities consisting of 100 species with functional contributions $\tilde{f}=\left({\tilde{f}}_{1},\ldots ,{\tilde{f}}_{100}\right)$ and abundances $a=\left(a_{1},\ldots ,a_{100}\right)$ via parameterized Dirichlet distributions (see Methods).

Introducing an increasing share of unclassified species with unknown functional contributions led to a higher distributional spread for all functional redundancy measures, reflecting increased random error and thus a higher uncertainty. The uncertainty was more pronounced with uneven distributions (α=1) than in scenarios with more even distributions (α=5) (Fig. 2C). Importantly, while for the sample taxon-based and abundance-based functional redundancy measure, no systematic error could be detected in this simulation setup, the true reference-based measure was systematically underestimated in the presence of unclassified species, which can be also theoretically explained (Supplementary Material, Attribute 5). As a result, systematic biases will be introduced when comparing reference taxon-based functional redundancies across populations with different shares of unclassified species. Thus, while the abundance-based and sample taxon-based measure suffer from increased random error with high numbers of unclassified species, the reference taxon-based measure will exhibit a systematic error underestimating the true functional redundancy.

### 4.3 Empirical applications using public metagenomic data

**Fig. 3:**
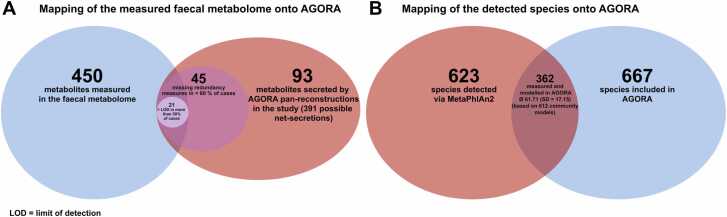
Overview of the utilised dataset. A, Mapping of the measured faecal metabolome onto the resource of genome-scale reconstructions (AGORA). B, Mapping of the detected species onto AGORA.

We calculated functional redundancy measures for 612 participants and 45 metabolites via employing COBRA community modelling using the faecal metagenomics data from Yachida et al. [1]. Table 1 and Fig. 3 provide an overview of the study, including characteristics of the utilised public dataset and summary statistics of the COBRA community models.

**Table 1:** Sample characteristics of the study.

	CRC Patients (n = 365)	Healthy controls (n = 247)	P-value
**Age, mean (SD)**	62.41 (9.90)	60.81 (12.59)	9.34e−02^a^
**BMI, mean (SD)**	22.95 (3.56)	22.68 (3.04)	0.30^a^
**Female, %**	39.18 %	46.15 %	9.52e−02^b^
**Species richness, mean (SD)**	69.75 (18.31)	64.70 (14.80)	1.87e−04^a^
**# Metabolites produced, mean (SD)**	209.25 (7.96)	208.48 (8.02)	0.25^a^
**# Reactions in community models, mean (SD)**	83310.11 (20725.58)	77674.06 (16850.53)	2.40e−04^a^

CRC = Colorectal cancer, SD = Standard deviation,

a Two-sided *t*-test,

b Fisher’s exact test

#### 4.3.1 Taxon-based and abundance-based measures of functional redundancy are complimentary

First, we analysed the empirical relations across the different operationalisations of functional redundancy introduced above (sample taxon-based vs. reference taxon-based vs. abundance-based, Table S4). Generally, R-squared values for sample taxon-based and reference taxon-based functional redundancy were substantial for most metabolites (R-squared > 0.4 for 41 of 45 metabolites). However, for taurine, cholic acid, L-cystine, and xanthosine, only moderate R-squared values (0.19–0.40) were observed, highlighting that reference and sample taxon-based functional redundancy are related but not equivalent across functions. The statistical relation is likely weakened for these four metabolites due to the strong difference between the number of species that could in theory produce a metabolite in the reference and the number of species with the function found in each sample (Table S4), introducing divergence between the reference-based measure and the sample-based measure. The intercept of the generally linear relation between reference- and sample taxon-based functional redundancy (Figure S1A) was logarithmically dependent both on the number of species capable of secreting the metabolite within the sample (Figure S1B) and the reference (Figure S1C), as expected given the mathematical properties of Eqn 1 and Eqn 2. In contrast, abundance-based functional redundancy measures correlated generally only little with taxon-based functional redundancy measures with the maximal R-squared being 0.15 (N-Acetyl-D-glucosamine, Figure S1D). In most cases, a slight negative correlation was observed, providing statistical evidence that the operationalisation of abundance-based and taxon-based functional redundancy capture two biologically distinct concepts. In essence, having many taxa being able to perform a function (high taxon-based functional redundancy) did not statistically correlate with having a high abundance of such taxa (high abundance-based functional redundancy). One explanation could be that having many similar species may lead to competition, limiting thereby each individual species in its abundance.

#### 4.3.2 Functional interdependency indices reveal extent of species-species interactions on metabolite secretions

**Fig. 4:**
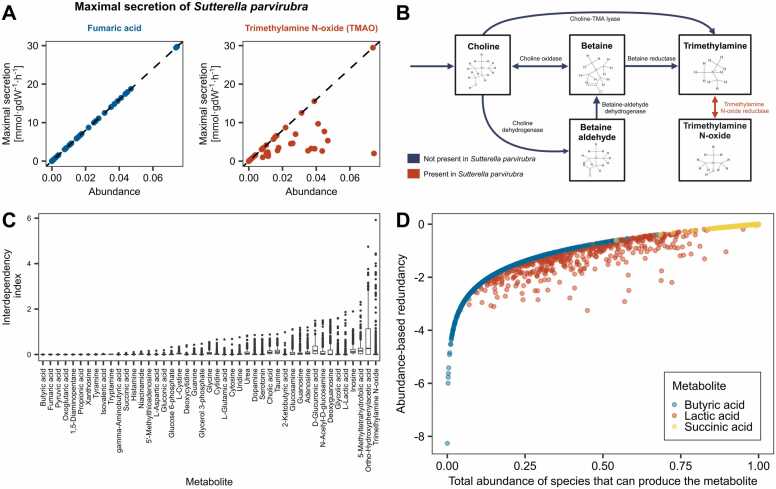
Results on functional interdependency using the CRC cohort. A, Maximal secretion potential (in mmol per (gram dry weight (gdW) * hour (h)) of *Sutterella parvirubra* of fumaric acid and trimethylamine N-oxide (TMAO) as a function of its abundance and linear dependency (dashed line). B, Pathway of AGORA reconstructions to produce Trimethylamine N-oxide, pathway in red is present in *Sutterella parvirubra*, whereas pathways in blue are not present in the reconstruction of *Sutterella parvirubra*. C, Boxplots of interdependency indices for each of the 45 metabolites, sorted by their interquartile range from lowest to highest. D, Scatterplot of the total abundance of species that can produce the metabolite to the abundance-based redundancy measure for butyric acid (blue), L-lactic acid (red) and succinic acid (yellow).

Recall that the functional interdependency index of a metabolite is zero if and only if the maximum secretion of the metabolite by a microbe is given by the same linear function of its abundance for all producing microbes in a sample. The higher the deviation from this proportionality attribute, the higher the functional interdependency index. In Fig. 4A, we demonstrate this concept using *Sutterella parvirubra* and its maximal *in silico* secretion of fumaric acid and trimethylamine N-oxide (TMAO). The maximal secretion of fumaric acid, a TCA cycle intermediate, by *Sutterella parvirubra* is strictly proportional to its abundance*,* independent of the community composition and thus the abundances of other species. In contrast, for TMAO, this strict proportionality is lost. The latter is the case if 1) a species does not contain the entire pathway for the production of a metabolite, or 2) metabolite availability in the production pathway is constrained by the presence or absence of other species in the community. Fig. 4B illustrates the union of metabolic pathways in AGORA reconstructions leading to TMAO production from choline. While *Sutterella parvirubra* encodes for a trimethylamine N-oxide reductase, enabling the conversion of trimethylamine (TMA) to TMAO, it lacks the enzymatic capacity to synthesize TMA from choline or betaine, rendering its TMAO production dependent on the presence of other species capable of TMA synthesis. The above species-interdependency pattern is not unique to *Sutterella parvirubra*, as fumaric acid exhibits lower functional interdependency indices than TMAO across all species (Fig. 4C), highlighting the broad relevance of metabolic cross-feeding interdependencies in TMAO production in comparison to other metabolites such as fumaric acid or butyric acid.

Importantly, the presence of functional interdependencies leads to lower abundance-based functional redundancy, given the same share of producing species in a community, as Fig. 4D shows for three common fermentation products (succinate, lactate, and butyrate). This is a plausible attribute. Consider, for example, a pathway that needs the cooperation of multiple species in comparison to a pathway that is present in each species alone. Losing one species will cause the loss of the function in the former case, while the function will be retained in the latter case. Thus, the functional interdependency index allows the mapping of the extent of interdependencies within a microbial community with regard to metabolic functions, as the TMAO example highlights.

#### 4.3.3 Rarity and functional interdependencies influence the relation between species diversity and functional redundancy

Given the importance of measures of species diversity in microbiome analysis and their theoretical relation to the proposed operationalisation of functional redundancy, we explored their relations to each other. In fact, in the specific scenario where every species can perform the function and there are no interdependencies between species, the vector of shares of functions, $\tilde{f}$, is equal to the vector of abundances, a (Supplementary Material, Attribute 1). In this case, the taxon-based measures are equivalent to Shannon entropy, except for a normalisation term. If this congruence would persist universally across all samples and functions, calculating functional redundancy following our operationalisations would carry no additional information in comparison to calculating the Shannon entropy as a measure of species diversity.

However, in the general case, the Shannon entropy and functional redundancy measures, as defined in this work, are not interchangeable. As rare functions impose a restriction in $\tilde{f}$, we expect that the statistical relationship between functional redundancy and species diversity weakens for rare functions, in particular if the total sum of abundances of species that can perform the function is low. As interdependencies introduce further variation in the divergence between a and $\tilde{f}$, we anticipate a diminished statistical relation between taxon-based functional redundancy and species diversity with increased interdependency.

Confirming the hypothesis that high species diversity leads to greater taxon-based functional redundancy, most but not all statistical associations between species diversity and functional redundancy measures were positive (Table S5). The strength of association was generally stronger for the reference taxon-based functional redundancy than for the sample taxon-based functional redundancy (Figure S2), which can be explained by varying normalisation terms across samples for the sample-based functional redundancy coefficient.

**Fig. 5:**
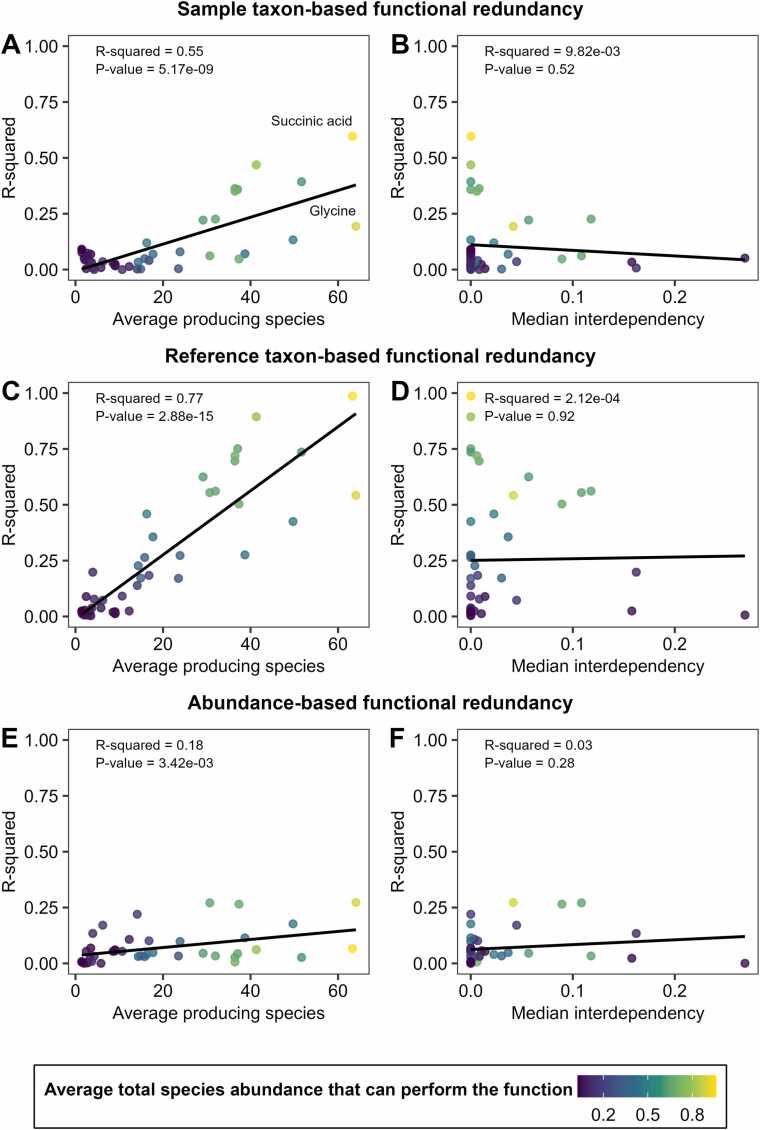
Scatterplots and regression lines for R-squared values between species diversity and measures of functional redundancy (Y-axis) against average number of producing species and the median functional interdependency index (X-axis) across all metabolites. In each scatterplot, the metabolites are coloured based on their average total species abundance that can perform the function. A, R-squared values (Y-axis, variance explained in sample taxon-based functional redundancy through species diversity) against average number of species with the function in a sample, and R-squared values (Y-axis, variance explained in sample taxon-based functional redundancy through species diversity) against median functional interdependency. B, R-Squared values (Y-axis, variance explained in reference taxon-based functional redundancy through species diversity) against average of species with the function in a sample, and R-squared values (Y-axis, variance explained in reference taxon-based functional redundancy through species diversity) against median functional interdependency. C, R-squared values (Y-axis, variance explained in abundance-based functional redundancy through species diversity) against average number of species with the function in a sample, and R-squared values (Y-axis, variance explained in abundance-based functional redundancy through species diversity) against median functional interdependency.

As supposed, the strength of the relationship was dependent on the commonness of a metabolic function across the sample, coupled to the total abundance of species that were capable of executing the function (Fig. 5A,C). Metabolite secretions characterised by a high number of producing species, such as succinate, with low functional interdependencies ($\mathrm{med}\left(I_{\mathrm{succinate}}\right)=1.42e-04$), demonstrated strong correlations between species diversity and taxon-based functional redundancy. For glycine, having comparable numbers and total abundances of producing species to succinate but higher interdependencies ($\mathrm{med}\left(I_{\mathrm{glycine}}\right)=0.04$), a lower R-squared value was observed. For functions that were rare and displayed a high level of functional interdependencies, the relation between species diversity and taxon-based functional redundancy was in tendency weaker. In line with our expectation, we did not observe high R-squared values between species diversity and measures of taxon-based functional redundancy for functions with high interdependencies (Table S5, Fig. 5B,D).

However, these arguments and statistical relations do not generally apply to abundance-based functional redundancy (Fig. 5E,F). While also negative in most cases, the statistical relationship between species diversity and abundance-based functional redundancy was generally weaker than for taxon-based measures and more often significantly reversed (Table S5). The latter effect highlights that high abundance-based functional redundancy can be achieved in ecologically non-diverse communities, where individual microbes expressing a certain function may dominate the community in terms of abundance. We also tested species richness, species evenness, and Simpson’s diversity as predictors, respectively, in analogous analyses, with species evenness and Simpson’s diversity yielding similar results, while the results for species richness differed in their overall structure (Table S6, Figure S3). In particular, R-squared values for species richness were much lower in general compared to Shannon diversity, Simpson diversity, and evenness (Tables S5, S6 and Figure S3), demonstrating that species richness in itself is only a weak predictor of functional redundancy.

#### 4.3.4 Measures of functional redundancy are associated with the host faecal metabolome

**Fig. 6:**
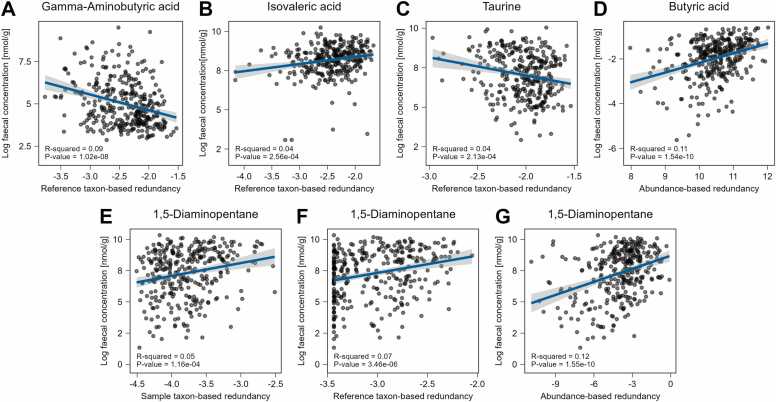
Scatterplots between measures of functional redundancy and log faecal concentrations of metabolites with fitted regression lines (blue) and confidence bands (grey). A, Scatterplot and fitted line between reference taxon-based functional redundancy of gamma-Aminobutyric acid and the log faecal concentration of gamma-Aminobutyric acid. B, Scatterplot and fitted line between reference taxon-based redundancy of isovaleric acid and the log faecal concentration of isovaleric acid. C, Scatterplot and fitted regression between reference taxon-based redundancy of taurine and the log faecal concentration of taurine. D, Scatterplot and fitted regression line between abundance-based redundancy of butyric acid and the log faecal concentration of butyric acid. E, Scatterplot and fitted regression line between sample taxon-based redundancy of 1,5-Diaminopentane and the log faecal concentration of 1,5-Diaminopentane. F, Scatterplot and fitted regression line between reference taxon-based redundancy of 1,5-Diaminopentane and the log faecal concentration of 1,5-Diaminopentane. G, Scatterplot and fitted regression line between abundance-based redundancy of 1,5-Diaminopentane and the log faecal concentration of 1,5-Diaminopentane.

Next, we analysed the statistical relations between measures of functional redundancy and host faecal metabolite concentrations for 21 of the 45 analysed metabolites (Fig. 3), utilising data from Yachida et al. [1]. In fully adjusted linear regression models, a total of 20 pairs achieved nominal significance with positive and negative associations. Seven associations remained significant after applying the Bonferroni correction for 63 tests (p < 7.94e-04, Table S7, Fig. 6). The top three hits belonged to the reference-based measure of gamma-Aminobutyric acid (GABA) (b = −0.85, 95 % CI: (-1.16, −0.54), p = 1.45e-07), the abundance-based measure of 1,5-Diaminopentane (cadaverine) (b = 0.32, 95 % CI: (0.22, 0.42), p = 3.94e-09), and the abundance-based measures of butyrate (b = 0.26, 95 % CI: (0.18, 0.34), p = 7.17e-10). Each of the three measures of functional redundancy of cadaverine was significantly positive correlated after multiple testing with its corresponding measured log faecal concentration (Fig. 6E-G). From the 21 tested metabolites, four were exclusively derived from the microbiome. Among these, three demonstrated a Bonferroni-corrected significant association with a redundancy measure. In contrast, only two metabolites that are co-secreted by the host showed similar associations, suggesting an enrichment of microbiome derived metabolites (Fisher’s exact test, p = 0.03, Table S7). As cadaverine and butyrate secretion are functions with close to zero functional interdependencies, higher abundance-based functional redundancies refer to communities with a large proportion of species that can produce the corresponding metabolite. Given that the average number of species able to secrete cadaverine in a community is relatively low (2.76, SD = 1.79), abundance-based functional redundancy measures close to zero, along with a high log faecal concentration of cadaverine, suggests the presence of a few key species that are crucial for cadaverine secretion. In contrast, as GABA and taurine are also produced by human pathways, the respective associations are not easily interpreted. In conclusion, the associations show that functional redundancy measures have correlates in the faecal host metabolome, in particular for microbiome-derived metabolites.

#### 4.3.5 Colorectal cancer affects the relation between functional redundancy and functional interdependency

To highlight the potential analysis routes incorporating clinical meta-data into the analysis of functional redundancy, we analysed the effect of colorectal cancer on measures of functional redundancy and functional interdependency. Notably, we observed a significant difference in species richness (number of species) in CRC patients compared to controls (b = 4.19, 95 % CI: (1.51, 6.86), p = 2.20e-03) and a marginally significant difference in species diversity (Shannon entropy) (b = 0.07, 95 % CI: (-2.62e-03, 0.14), p = 0.06). A total of 24 functional redundancy-measures, most of them belonging to the reference taxon-based measure (16), and eight functional interdependency indices showed nominal significance (Table S8). After adjusting for 180 tests with the Bonferroni method, the reference taxon-based functional redundancy of pyruvate (b = 0.13, 95 % CI: (0.06, 0.20), p = 2.23–04, Table S8) was the only measure achieving statistical significance (p < 2.78e-04). Despite none of the interdependency measures being significant after multiple testing correction, all but one nominally significant associations demonstrated positive coefficients. Indeed, the log-scaled global functional interdependency index revealed a positive association between global functional interdependency and CRC (b = 1.16, 95 % CI: (0.15, 2.17), p = 0.02), indicating that CRC microbiomes display higher levels of functional interdependencies.

#### 4.3.6 Inflammatory bowel disease impacts functional redundancy beyond species diversity

**Fig. 7:**
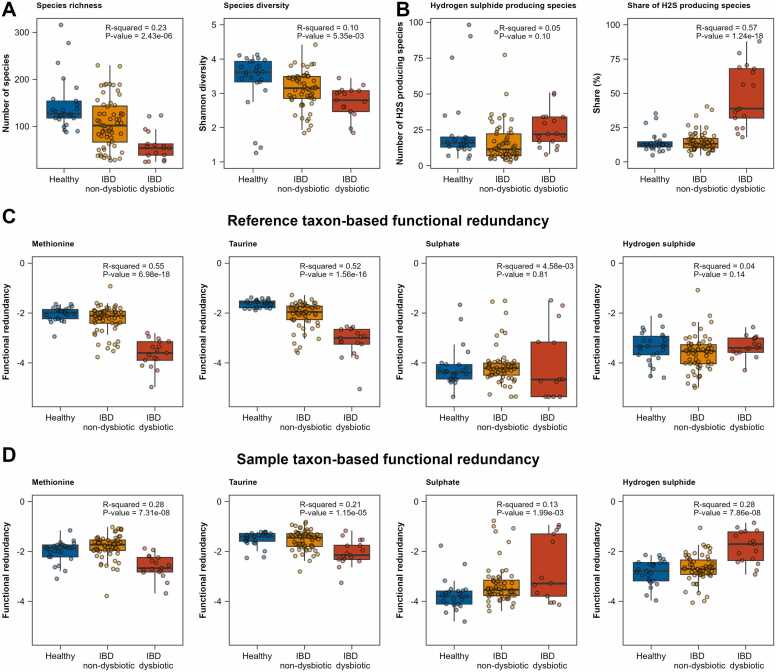
Boxplots illustrating ecological diversity, hydrogen sulphide-specific outcomes, and measures of functional redundancy in healthy, IBD non-dysbiotic, and IBD dysbiotic samples. A, Species richness and species diversity. B, Number and share of hydrogen sulphide producing species. C, Reference taxon-based measures of functional redundancy for metabolites implicated in sulphur metabolism. D, Sample taxon-based measures of functional redundancy for metabolites implicated in sulphur metabolism. P-values are derived from ANOVA analyses without incorporating any contrasts.

To showcase a situation of a disease with well-evidenced influence on species diversity, we utilised an existing inflammatory bowel disease (IBD) study, from which we analysed 102 microbiome community models (n = 17 dysbiotic IBD, n = 60 non-dysbiotic IBD, n = 25 healthy controls). We computed redundancy measures for 161 metabolites (Table S3). As expected, species richness (ANOVA: F = 14.78, p = 2.43e-06) and Shannon diversity (ANOVA: F = 5.52, p = 5.35e-03) were clearly lower in the IBD conditions (Fig. 7A).

After Bonferroni correction across 483 tests (161 metabolites × 3 redundancy measures), 122 metabolites showed significant differences (p < 1.04e-4) in at least one redundancy measure (Table S9). Most of the significant changes were observed in the reference-based measure (87 metabolites), with all but two showing a reduction in redundancy for IBD patients compared to controls (Table S10). Even when adjusting for the loss of species diversity, 121 associations with the disease status (healthy vs. dysbiotic IBD vs. non-dysbiotic IBD) were significant after multiple testing correction, demonstrating that functional redundancy measures carry information beyond species diversity (Table S11). Generally, the sample-based measure showed lower effect sizes than the reference-based measure (Fig. 7D), which could be due to reduced comparability of sample-based measures across communities with strong differences in species richness.

For illustration, we focus on sulphur metabolism, as sulphur metabolism has been implicated in the pathophysiology of IBD [58], [59], [60], [61]. Reference taxon-based measures reveal substantial losses in functional redundancy in IBD patients compared to controls, particularly for methionine (b = −0.90, 95 % CI: (-1.14, −0.66), p = 3.45e-11), and taurine (b = −0.99, 95 % CI: (-1.22, −0.76), p = 1.97e-13). Remarkably, reference taxon-based taurine functional redundancy measures perfectly separated between dysbiotic IBD cases from healthy controls (Fig. 7C), indicating that taurine functional redundancy could be a dysbiosis biomarker in IBD. However, other important sulphur compounds, such as sulphate and hydrogen sulphide, did not show similar declines (Fig. 7C). Interestingly, despite the loss in species richness, no decrease in hydrogen sulphide-producing species was observed, leading to an increased proportion of hydrogen sulphide-producing species in IBD patients (Fig. 7B). As a consequence, the reference-based measure for hydrogen sulphide did not show significant differences of IBD patients compared to controls, while the sample-based measure even increased. This result highlights how the functional changes in IBD microbiomes driven by a strong loss in species diversity can be fine-mapped by examining functional-specific changes that are not captured by species diversity alone.

## 5 Discussion

Species diversity of the human gut microbiome is thought to contribute to human health and disease through functional redundancy. Here, we introduce and analyse an information-theoretic approach to functional redundancy that enables the function-specific quantification of functional redundancy. The herein introduced operationalisation of functional redundancy is markedly different from previous operationalisations based on community-level multi-trait functional dissimilarity measures [28], [46], [47], [56], [57], which are not easily interpreted and meaningfully applied to the analysis of functional redundancy of individual traits.

To clarify the scope, we defined functional redundancy in three different contexts. With the sample taxon-based functional redundancy, the quantification is coupled to the species prevalent in a concrete sample, whereas reference taxon-based functional redundancy is calculated by taking all species into account that could perform a function in theory based on references such as AGORA [38] or AGORA2 [37]. Abundance-based functional redundancy, on the other hand, is defined on the abundance of organisms that could perform a function. The three operationalisations are statistically interrelated (Fig. S1, Table S4) but neither statistically, nor conceptually redundant to each other, since they capture different distributional aspects of functions across microbiomes and reflect different ways to understand functional redundancy.

Which of the operationalisations should be preferred depends on the research question and study design aspects. For example, in a situation without a suitable reference or in situations with highly variable amounts of unclassified species, the sample-based measure could be superior to the reference-based measure, as the latter suffers from systematic bias from unclassified species. In contrast, the sample-based measure, due to its dependence on the species richness, may suffer from less comparability across populations with equal shares of unclassified species but strong differences in species richness.

Our approach also opened up the possibility to derive measures of functional interdependency across species. The degree of species-species interactions has been challenging to determine [62], [63], [64], [65], even within relatively simplified and controlled modelling environments. Our operationalisation gives a computationally feasible solution to assess the extent of functional interdependencies on metabolite secretions. The analysis shows, for example, that microbial TMAO production is a pathway with strong species-species interdependencies. This result is of potential clinical interest, since TMAO has been implicated in atherosclerosis and chronic kidney disease [66], [67], [68], [69], [70].

From an information-theoretic perspective, the interdependency index reflects the extent of information encoded within species abundances when modelling a function of a microbial community using their abundances. Similar to the concept of conditional entropy H(Y|X) of two random variables that is zero if and only if Y is completely determined by X, the functional interdependency index is zero if and only if the functional output of each species is a linear function of its abundance. Therefore, the functional interdependency index may be used in investigating causal structures of microbiomes [44], [71], [72] as, for example, in structural equation models [73], [74], as conditional entropy is already used for identifying causal relations [75], [76], [77]. However, the interdependency index does not capture all aspects of species-species interdependencies as ecological relationships such as symbiosis or competition cannot be inferred from the measure. It also does not capture other aspects important to species-species relations such as host physiology or dietary behaviour.

Analysing the relation between species diversity and measures of functional redundancy, we observed that for rare functions and functions with high interdependencies, functional redundancy is seemingly determined by the presence and abundance of *specific* microbes rather than by the species diversity in general. In contrast, with common functions characterised by low functional interdependencies, high species diversity will often directly lead to high functional redundancy. These insights may help understanding the observed relation between high species diversity in the gut microbiome and human health [78], [79], [80], [81]. If functional redundancy is the mediating factor between species diversity and human health, this would point to relatively common functions with low species-species functional interdependencies such as butyrate production, for example. Thus, the concepts allow for the targeted investigation of individual metabolic functions mediating between human health and microbial diversity.

In this vein, our results highlight evidence for increased species-species functional interdependencies in CRC microbiomes. This finding may reflect less functional stability in CRC microbiomes, contributing thereby to CRC specific dysbiosis. We also demonstrated that the introduced measures have correlates in the host metabolome, in particular for microbiome-specific metabolites such as cadaverine and butyrate. In the case of butyrate, an important short chain fatty acid and nutrient for colonocytes [82], [83], [84], [85], we could show that higher functional redundancy is associated with higher faecal butyrate levels. Noteworthy though, functional redundancy in general is not necessarily indicative of “high function”, since functional redundancy describes a potential to retain a function under perturbation rather than the absolute level of a function. In general, as the introduced operationalisation is based on a projected potential to retain a function under biomass loss in contrast to an observed capacity, results from such analyses have hypothetical character and thus are warranting further validations.

In a further example, our results demonstrated that the introduced redundancy measures can fine-map the changes in IBD-associated microbiomes, which are generally associated with a loss of species diversity [86], [87], [88], [89]. While IBD mostly led to reduced functional redundancy across a wide range of functions in our analysis, certain key functions such as hydrogen sulphide production did not suffer from a loss in functional redundancy despite decreased sulphur metabolism diversity in IBD [3]. Hydrogen sulphide is a pro-inflammatory metabolite [90], [91], generated during the breakdown of sulphur-containing amino acids [92]. It plays a pivotal role in modulating inflammatory responses through the activation of immune cells and the production of pro-inflammatory cytokines [93], [94]. Thus, while functional redundancy is overall reduced in IBD, communities stay remarkably functionally redundant with regards to hydrogen sulphide production with possible clinical implications.

Several limitations of our approach have to be kept in mind. The information theoretic approach to functional redundancy can, in principle, be applied to any ecosystem where quantitative data on species-specific contributions to a single function or an aggregated function (e.g., based on pathways) along with their abundances exist. However, species-specific contributions are often lacking. For example, functional profiles derived from 16S rRNA gene sequencing data are commonly utilised in tools such as PICRUSt2 [95], [96], [97]. In contrast to our approach, such methods based on aggregated gene abundances on the community level do not provide a suitable basis for the introduced information theoretic approach. Furthermore, our definitions are not specifically designed to capture aspects of functional redundancy as represented, for example, in time-series data. Future research may show whether suitable and fruitful generalisations to longitudinal settings within the outlined framework of information theoretic measures can be formulated. Using constraint-based reconstruction and analysis (COBRA) to model the maximum secretion fluxes of species that form a microbial community comes with several limitations that should be noted. The applied modelling relies on the simulation of fluxes (not concentrations) under the steady state assumption, meaning that the input for the functional redundancy measures are based on simplifications and may not hold in situations of dynamic challenges (e.g., antibiotics intake, changes in diet). Moreover, the modelling approach neglected the spatial structure of the gut microbiome. Microbiome functions might vary in different biofilms [98], [99], compartments [100] or host characteristics which we did not model. However, ecosystem compartmentalisation can be integrated by calculating functional redundancy in a compartmentalised way if sufficient information about the spatial distribution of species is available. Flux calculations rely on correct representation of the metabolic capacities of microbial species in the knowledge-base. However, certain pathways and species are better researched than others and the thereby introduced biases represent a core limitation of knowledge-based modelling techniques such as COBRA. Furthermore, the introduced measures of functional redundancy are constructed on relative abundances and relative functional shares. While in the case of microbiome data, compositional data is normally the data-type available, it is plausible that important information on functional redundancy is coded in absolute abundances and absolute function values.

## 6 Conclusions

Formalising measures of metabolic functional redundancy via information theoretical concepts offers a promising avenue for understanding microbial community functions in relation to microbiome diversity. Building on the concept of relative entropy, we introduced information-theoretic operationalisations not only of functional redundancy in various contexts, but also of functional interdependency within microbiomes. Our work shows 1) that the relation between microbiome diversity and patterns of functional redundancy is dependent on the attributes of the function of interest, 2) microbiome functional redundancy has correlates in the host’s metabolome, and 3) can reveal hidden functional differences across medical conditions as exemplified by the analysis of CRC and IBD microbiomes. We believe that the introduced measures can serve as a valuable complement to conventional species diversity measures, providing a nuanced perspective on microbial community functions and their interdependencies with a wide range of applications in microbiome research.

## Funding

This work was funded by the 10.13039/501100001659Deutsche Forschungsgemeinschaft (DFG, GermanResearch Foundation) Project-ID 499552394 – SFB 1597 and HE9198/1-1 to J.H. A.H. received funding from the Agence Nationale de la Recherche (ANR) under the decree 2021-1710.

## CRediT authorship contribution statement

**Fässler Daniel:** Writing – review & editing, Writing – original draft, Visualization, Software, Methodology, Formal analysis, Data curation, Conceptualization. **Hertel Johannes:** Writing – review & editing, Writing – original draft, Supervision, Methodology, Formal analysis, Conceptualization. **Heinken Almut:** Conceptualization, Data curation, Software, Writing – review & editing.

## Declaration of Competing Interest

The authors declare that they have no known financial interests or personal relationships that could have influenced the work presented in this paper.

## Data Availability

The raw data of the utilised colorectal cancer study can be found in the stem publication and is available in [1]. The raw IBD study can be found in [2]. Utilised AGORA reconstructions can be found on https://github.com/VirtualMetabolicHuman/AGORA/. Scripts to generate microbiome community models for the CRC study and individual maximum secretion fluxes for the CRC study can be found on https://github.com/SysPsyHertel/CodeBase/tree/main/Scripts_Faessler_Functional_Redundancy. The scripts for running microbiome community models and maximum secretion fluxes for the IBD study can be found on https://github.com/ThieleLab/CodeBase/tree/master/Analysis_Heinken_npj_Syst_Biol_Appl_2021 and is explained in [3]. The R package for computing measures of functional redundancy can be found on https://github.com/SysPsyHertel/FunRed The generated individual maximum secretion fluxes for the CRC study can be found in the Table S1. The computed functional redundancy and functional interdependency measures for the IBD and CRC study are included in this published article (Table S2 (CRC study) and Table S3 (IBD study)). All scripts and code utilised to generate the results in this work based on the generated individual maximal secretion fluxes, including simulations can be found on https://github.com/SysPsyHertel/CodeBase/tree/main/Scripts_Faessler_Functional_Redundancy. The Graphical Abstract (https://BioRender.com/tboy2bh), Figure 1 (https://BioRender.com/d2wx0pg), and Figure 4B (https://BioRender.com/eecp36i) were created in BioRender.
