# Supplementary material for: Characterising functional redundancy in microbiome communities via relative entropy
Source: Comput Struct Biotechnol J. 2025 Mar 12;27:1482–97. doi: 10.1016/j.csbj.2025.03.012 (PMC12013412; doi:10.1016/j.csbj.2025.03.012)
Supplement: Supplementary file 1 — Supplementary material [file mmc1.docx]

**Supplementary Material**

**Content**

[**1** **Theoretical definitions** 1](#_Toc194574316)

[**2** **Attributes** 2](#_Toc194574317)

[**3** **Operationalisation of taxon-based functional redundancy, abundance-based functional redundancy, and functional interdependency via maximum secretion fluxes** 5](#_Toc194574318)

[**4** **Toy example** 7](#_Toc194574319)

[**5** **Supplementary Figures** 9](#_Toc194574320)

# **Theoretical definitions**

**Kullback-Leibler divergence**

Let $P$ and $Q$ be two discrete probability distributions. We define $p\left( x \right)=P(\left\{ x \right\})$ and $q\left( x \right)=Q(\left\{ x \right\})$ over a set $X$. The Kullback-Leibler divergence or relative entropy between $P$ and $Q$ is defined to be:

|  | $D_{KL}\left( P\left\Vert Q \right. \right)=\sum_{x\in X} p\left( x \right)\log\left( \frac{p\left( x \right)}{q\left( x \right)} \right).$ | (1) |
| --- | --- | --- |

Note that we sum only over all non-trivial $p\left( x \right),$ $x\in X$. For all $x\in X$, if $q\left( x \right)=0$, it must apply that $p\left( x \right)=0$ (absolute continuity). It can be written in different forms:

$D_{KL}\left( P\left\| Q \right. \right) =\mathbb{E}_{P}\left[ \log\left( \frac{p\left( X \right)}{q\left( X \right)} \right) \right]=\mathbb{E}_{P}\left[ \log p\left( X \right) \right]-\mathbb{E}_{P}\left[ \log q\left( X \right) \right]=H\left( P,Q \right)-H(P)$, where

|  | $H\left( P,Q \right)=\sum_{x\in X} p\left( x \right)\log\left( \frac{1}{q\left( x \right)} \right)$ | (2) |
| --- | --- | --- |

is the cross-entropy of $P$ and $Q$ and

|  | $H\left( P \right)=H(P,P)= \sum_{x\in X} p\left( x \right)\log\left( \frac{1}{p\left( x \right)} \right)$ | (3) |
| --- | --- | --- |

is the entropy of $P$.

# **Attributes**

Let $\boldsymbol{f}=(f_{1},\ldots,f_{n})$ be a quantitative vector of realisations of the random vector $\boldsymbol{F}$, understood as the contributions to a function belonging to a community of $n$-species. We denote $\tilde{\boldsymbol{f}}=(\tilde{f}_{1},\ldots,\tilde{f}_{n})$ as the respective vector of relative frequencies of $\boldsymbol{f}$, which is understood as a distribution expressed in probabilities. Furthermore, let $J=\left\{ i:\tilde{f}_{i}>0 \right\}$ be the indices of the shares that are greater zero. Let $\boldsymbol{U}_{n}$ be a uniform distribution, and let $\boldsymbol{a}\boldsymbol{=}(a_{1},\ldots,a_{n})$ be a vector corresponding to the random vector $\boldsymbol{A}$, representing the abundance of the $n$-species in the sample, i.e., $\sum_{i=1}^{n} a_{n}=1.$ Furthermore, let $m$ be the number of species in the reference set that can theoretically perform the function.

**Attribute 1:**

Let all components of $\boldsymbol{f}$ that are greater zero be the same linear function of the corresponding components of $\boldsymbol{a}$**,** that is, if $f_{i}=ba_{i}$ for all $i\in J$, where $b$ is a constant (no interdependencies exist), then the abundance-based functional redundancy measure simplifies to $\log\left( \sum_{i\in J} a_{i} \right)$, which is the log-scaled total sum of the abundances/shares of species that can perform the function. In the scenario of $\left| J \right|=n$, where every species can perform the function and there are no interdependencies between species, the vector of shares of functions, $\tilde{\boldsymbol{f}}$, is equal to the vector of abundances, $\boldsymbol{a}$.

**Proof:**

In the general case:

$R_{Abundance}={-D}_{KL}\left( \tilde{\boldsymbol{f}}\left\| \boldsymbol{a} \right. \right)=-\sum_{i\in J} \frac{ba_{i}}{b\sum_{u\in J} a_{u}}\log\left( \frac{\frac{ba_{i}}{b\sum_{u\in J} a_{u}}}{a_{i}} \right)=\log(\sum_{u\in J} a_{u})$.

If every species can perform the function and no interdependencies exist, it holds that $\boldsymbol{f}=(ba_{1},\ldots,ba_{n})$ and therefore $\tilde{\boldsymbol{f}}\boldsymbol{=}\left( \tilde{f}_{1},\ldots,\tilde{f}_{n} \right) \boldsymbol{=}\frac{1}{\sum_{i=1}^{n} ba_{i}}\left( ba_{1},\ldots,ba_{n} \right)\boldsymbol{=}\frac{b}{\sum_{i=1}^{n} ba_{i}}\left( a_{1},\ldots,a_{n} \right)\boldsymbol{=}\left( a_{1},\ldots,a_{n} \right)$, because of $\sum_{i=1}^{n} a_{i}=1.$ □

**Attribute 2:**

The taxon-based functional redundancy of a quantitative function of $n$-species and a reference set of $m$-species is minimal, if and only if one species can perform the function. Then, it holds that $R_{Taxon}=-log \left( n \right)$ when using the sample-based measure and $R_{Taxon}=-log \left( m \right)$ when using the reference-based measure. The sample-based measure is maximal if and only if all species in the sample perform the same amount of function, meaning that $\tilde{f}_{i}=\frac{1}{n}$, for all $i\in\{1,\ldots,n\}$. The reference-based measure is maximal, if and only if all species in the reference are in the sample and perform the same amount of function, meaning that $\tilde{f}_{i}=\frac{1}{m}$, for all $i\in\{1,\ldots,m\}$. In both cases, the maximal taxon-based measure is zero.

**Proof:**

Using equation (1) from the manuscript, it implies for the sample-based measure that:

$R_{Taxon}={-D}_{KL}\left( \tilde{\boldsymbol{f}}\left\| \boldsymbol{U}_{n} \right. \right)={H\left( \tilde{\boldsymbol{f}} \right)-H}_{\max}\left( \tilde{\boldsymbol{f}} \right)=H\left( \tilde{\boldsymbol{f}} \right)-\log(n)$. From the properties of entropy, $H(.)$, it follows that the term $H\left( \tilde{\boldsymbol{f}} \right)$ minimal if there exists only one $i\in\{1,\ldots,n\}$ such that $\tilde{f}_{i}=1$. In that case, $H\left( \tilde{\boldsymbol{f}} \right)$ is zero, which implies that $R_{Taxon}=-\log(n)$. The maximal entropy of $\tilde{\boldsymbol{f}}$ is achieved, if $\tilde{\boldsymbol{f}}$ corresponds to an uniform distribution, $U_{n}$. In that case, $H\left( \tilde{\boldsymbol{f}} \right)=\log(n)$ and $R_{Taxon}=0$. The same argument holds when using the reference-based measure. □

**Attribute 3:**

Let $n$ be the number of species present in a community. If the community is uniformly distributed, then the sample taxon-based and abundance-based measures are equal.

**Proof:**

$$R_{Abundance}={-D}_{KL}\left( \tilde{\boldsymbol{f}}\left\| \boldsymbol{a} \right. \right)=-\sum_{i\in J} \tilde{f}_{i}\cdot log\left( \frac{\tilde{f}_{i}}{a_{i}} \right)=-\sum_{i\in J} \tilde{f}_{i}\cdot log\left( {n\tilde{f}}_{i} \right)$$

$=-\sum_{i\in J} \tilde{f}_{i}\cdot log\left( \tilde{f}_{i} \right)-\log(n) =R_{taxon}$ (sample-based). □

**Attribute 4:**

Let $\boldsymbol{f}=(f_{1},\ldots f_{n})$ be a function with functional shares $\tilde{\boldsymbol{f}}=\left( \tilde{f}_{1},\ldots,\tilde{f}_{n} \right)$, for which we have computed $R_{Taxon}$. Let $p$ be the number of newly introduced species to the community, which were previously unknown, with their corresponding functional contributions $\left( f_{n+1},\ldots,f_{n+p} \right)$. These represent $p$ newly introduced species that are now present in both the sample and the reference. If the contributions of the $p$-unknown species are zero, then $R_{Taxon}^{'}-R_{Taxon}=\log\left( \frac{n}{n+p} \right)$ (sample-based), and similarly, $R_{Taxon}^{'}-R_{Taxon}=\log\left( \frac{m}{m+p} \right)$ (reference-based).

**Proof:**

It holds for the sample taxon-based measure (before introducing $p$ species to the community):

$R_{Taxon}={-D}_{KL}\left( \tilde{\boldsymbol{f}}\left\| \boldsymbol{U}_{n} \right. \right)={H\left( \tilde{\boldsymbol{f}} \right)-H}_{\max}\left( \tilde{\boldsymbol{f}} \right)=\sum_{i\in J} \tilde{f}_{i}\log\left( \tilde{f}_{i} \right)-log \left( n \right)$. After adding $p$-species that were previously unknown with zero functional contributions, only the maximal functional entropy changes from the term of $\log(n)$ to $\log(n+p)$, meaning that $R_{Taxon}^{'}-R_{Taxon}=\log\left( n \right)-\log\left( n+p \right)=\log\left( \frac{n}{n+p} \right)$. The same argument holds when using the reference taxon-based measure. □

**Attribute 5:**

The taxon-based functional redundancy of a quantitative function of $n$-species, knowing that $n>k$-species in the sample can perform the function (i.e., $|J|=k$), is maximal when $\tilde{f}_{i}= \tilde{f}_{j}$ for all­­ $i,j\in J$. In this case, $R_{Taxon}=\log\left( \frac{k}{n} \right)$ when using the sample-based measure, and $R_{Taxon}=\log\left( \frac{k}{m} \right)$ when using the reference-based measure.

**Proof:**

Let $J^{C}=\left\{ i:f_{i}=0 \right\}\neq\emptyset$. We need to show for the sample taxon-based functional redundancy operationalisation, that $\max_{\left( \tilde{f}_{1},\ldots,\tilde{f}_{n} \right)} {-D}_{KL}\left( \tilde{\boldsymbol{f}}\left\| \boldsymbol{U}_{n} \right. \right) =\log\left( \frac{\left| J \right|}{n} \right)$, where $\tilde{f}_{i}=0$, for all $i\in J^{C}$. It follows that:

$${-D}_{KL}\left( \tilde{\boldsymbol{f}}\left\| \boldsymbol{U}_{n} \right. \right)=-\sum_{i\in J} \tilde{f}_{i}\log\left( \tilde{f}_{i} \right)-\log\left( n \right).$$

As the first term represents entropy ($H(.)$) on the set $J$, that is maximal, if $\tilde{f}_{i}= \tilde{f}_{j}$ for all­­ $i,j\in J$. The term $log(n)$ is fixed and always greater than the left term, as $J^{C}\neq\emptyset$ and $n>\left| J \right|$. Therefore, it holds that:

$\max_{(\tilde{f}_{1},\ldots,\tilde{f}_{n})} {-D}_{KL}\left( \tilde{\boldsymbol{f}}\left\| \boldsymbol{U}_{n} \right. \right)=\log\left( \left| J \right| \right)-\log\left( n \right)=\log\left( \frac{\left| J \right|}{n} \right)=\log\left( \frac{k}{n} \right)$, where $\tilde{f}_{i}=0$, for $i\in J^{C}$.

Thus, the upper bound, representing the maximal taxon-based functional redundancy, when $k$ species can perform the function of interest out of $n>k$ is $\log\left( \frac{k}{n} \right)$. □

# **Operationalisation of taxon-based functional redundancy, abundance-based functional redundancy, and functional interdependency via maximum secretion fluxes**

Let $C$ be a set of microbial communities. Each community $c\in C$ is defined by a vector of microbial abundances $\boldsymbol{a}_{c}\in\left[ 0,1 \right]^{L}$, where $L$ denotes the number of species in the union of all communities, which can be mapped onto AGORA that we denote as a set of microbial reconstructions, $R$. For each microbial community, we define an associated COBRA community which consists of a set of (pan-)genome scale reconstructions, $R_{c}\subseteq R$, where $|R_{c}|\leq L$ for each $c\in C$ and associated constraints, such as diet constraints or coupling constraints. Furthermore, let $M$ be a set of metabolites. We can then define, for each $m\in M$, the set $R_{m}\subseteq R$ of reconstructions, that can secrete metabolite $m$. Let $IEX_{c,m,l}\geq0$ be the individual maximum secretion flux of COBRA community model $c$, metabolite $m$ and for microbe $l\in\{1,\ldots,L\}$. If $R_{c}\cap R_{m}\neq\emptyset$, then COBRA community model $c$ has at least one reconstruction that can secrete metabolite $m$, and this enables us to compute the individual maximum secretion profile for all $IEX_{c,m,l}$, $c\in C, m\in M, l{\in R}_{c}\cap R_{m}$. Otherwise, if no species in a COBRA community model $c$ can secrete$m\in M$, then we define $IEX_{c,m,l}=0,$ i.e., for all $l{\notin R}_{c}\cap R_{m}$. Note that the maximum secretion fluxes are absolutely continuous by design, as a species that is not present in a community cannot perform a function. We can now define the sample taxon-based secretion vector for each COBRA community model $c$ and metabolite $m$:

|  | $\boldsymbol{f}_{sample_{c,m}}=\left( IEX_{c,m,l} \right)_{l\in R_{c}},$ | (4) |
| --- | --- | --- |

which represents a vector of length $|R_{c}|$, consisting of all maximum secretion capacities of metabolite $m$ for each species present in the COBRA community model $c$. We set $IEX_{c,m,l}=0$, if $l\notin R_{m}$, meaning, that the genome-scale reconstruction $l$ is in the sample but has no exchange reaction. Note that $IEX_{c,m,l}=0$ is not a sufficient condition for $l\notin R_{m}$. It may also be the case that a reconstruction has an exchange reaction but is not able to secrete it in a COBRA community model. Canonically, we define the reference-based secretion vector for each COBRA community model $c\in C$ and metabolite $m\in M$:

|  | $\boldsymbol{f}_{reference_{c,m}}=\left( IEX_{c,m,l} \right)_{l\in R_{m}},$ | (5) |
| --- | --- | --- |

where we set $IEX_{c,m,l}=0$, if $l\notin R_{c}$, meaning the genome-scale reconstruction, $l$ is not in the sample. The reference-based secretion, $\boldsymbol{f}_{reference_{c,m}}$ is a vector of length $|R_{m}|$, consisting of all maximum secretion capacities of metabolite $m$ in COBRA community $c$. Note that every non-zero entry in $\boldsymbol{f}_{sample_{c,m}}$ is a component in $\boldsymbol{f}_{reference_{c,m}}$ and vice versa. What differs is the number of non-zero elements. With above conventions, we define the sample taxon-based functional redundancy of a COBRA community model $c\in C$ and metabolite $m\in M$, containing $|R_{c}|$ mapped-microbes, as the negative relative entropy between the normalised ${\tilde{\boldsymbol{f}}}_{sample_{c,m}}$ and a discrete uniform distribution, $\boldsymbol{U}_{|R_{c}|}$:

|  | ${R_{Taxon}}_{c,m}={-D}_{KL}\left( \frac{1}{\sum_{i\in R_{c}} {IEX}_{c,m,i}} \left( IEX_{c,m,l} \right)_{l\in R_{c}}\left\Vert\boldsymbol{U}_{\vert R_{c}\vert} \right. \right),$ | (6) |
| --- | --- | --- |

Consequently, we define the reference taxon-based functional redundancymeasure of each COBRA community model $c\in C$ and metabolite $m\in M$, using the $|R_{m}|$ species in the reference that can secrete a metabolite in the AGORA resource across the study to calculate the Kullback-Leibler divergence:

|  | ${R_{Taxon}}_{c,m}={-D}_{KL}\left( \frac{1}{\sum_{i\in R_{c}} {IEX}_{c,m,i}} \left( IEX_{c,m,l} \right)_{l\in R_{m}}\left\Vert\boldsymbol{U}_{\vert R_{m}\vert} \right. \right)$. | (7) |
| --- | --- | --- |

To operationalise the abundance-based functional redundancy, let $\boldsymbol{a}_{c}\in\left[ 0,1 \right]^{|R_{c}|}$ be the individual microbial abundance vector in community model $c\in C.$ Then, we define abundance-based functional redundancy, ${R_{Abundance}}_{c,m}$ of each COBRA community model $c\in C$ and metabolite $m\in M$ as

|  | ${R_{Abundance}}_{c,m}={-D}_{KL}\left( \frac{1}{\sum_{i\in K_{c,m}} {IEX}_{c,m,i}} \left( IEX_{c,m,l} \right)_{l\in R_{c}}\left\Vert\boldsymbol{a}_{c} \right. \right)$. | (8) |
| --- | --- | --- |

Finally, we operationalise the interdependency index based on maximal secretion potentials. Let $V_{c,m}$ be the set of microbes, for which $IEX_{c,m,l}>0$. Now, we define $\left( IEX_{c,m,l} \right)_{l\in V_{c,m}}$as the secretion vector for model $c$ and metabolite $m$ that are greater zero. Let $a_{c,m,l}$ be the corresponding abundance of microbe $l\in V_{c,m}$of community $c$ and metabolite $m$ and $\boldsymbol{a}_{c,m}=\left( a_{c,m,l} \right)_{l\in V_{c,m}}$ the vector of abundances of species that correspond to the maximum secretions, $\left( IEX_{c,m,l} \right)_{l\in V_{c,m}}$. We define the interdependency index $I_{c,m}$ of the metabolite $m$ corresponding to the model $c$ as the relative entropy between the vector of shares of $\left( IEX_{c,m,l} \right)_{l\in V_{c,m}}$and the normalised vector of $\boldsymbol{a}_{c,m}$:

|  | $I_{c,m}={-D}_{KL}\left( \frac{1}{\sum_{i\in V_{c,m}} {IEX}_{c,m,i}} \left( IEX_{c,m,l} \right)_{l\in V_{c,m}}\left\Vert\frac{1}{\sum_{i\in V_{c,m}} a_{c,m,i}}\boldsymbol{a}_{c,m} \right. \right)$. | (9) |
| --- | --- | --- |

The global functional interdependency index for a community model $c\in C$, consists of the median of metabolites $m\in M$:

|  | $I_{g,c}=\mathrm{med}{({\{I}_{c,m}\}}_{m\in M})$. | (10) |
| --- | --- | --- |

# **Toy example**

For the exemplification of the proposed operationalisation, consider the following toy example, a system consisting of $n=5$ species, $\{S_{1},{\ldots,S}_{5}\}$, with abundances $\boldsymbol{a}=\left( a_{1},a_{2},a_{3},a_{4},a_{5} \right)=\left( 0.2,0.1,0.05,0.05,0.6 \right)$ and associated paired feature vector of metabolic functions (here, the maximal secretion profile expressed as shares of the total community secretion) $\tilde{\boldsymbol{f}}=\left( \tilde{f}_{1},\tilde{f}_{2},\tilde{f}_{3},\tilde{f}_{4},\tilde{f}_{5} \right)=\left( 0.8,0.1,0.05,0.05,0 \right)$ (Fig. 1A). In this toy example, one species ($S_{5}$) cannot secrete the metabolite of interest (according to the reference), while three other species ($S_{6}$,$S_{7}$, $S_{8}$) in the reference can potentially perform the metabolic function. Thus, ${\tilde{\boldsymbol{f}}}_{ref}=\left( \tilde{f}_{1},\tilde{f}_{2},\tilde{f}_{3},\tilde{f}_{4},\tilde{f}_{6},\tilde{f}_{7},\tilde{f}_{8} \right)=\left( 0.8,0.1,0.05,0.05,0,0,0 \right).$Note that we do not include a value for $S_{5}$ in ${\tilde{\boldsymbol{f}}}_{ref}$, as species $S_{5}$ cannot secrete the metabolite of interest in this toy example. The basis for the reference taxon-based feature vector is a vector derived from species in the reference that can perform the function. We will now first discuss the measures of taxon-based functional redundancy, followed by the measure of abundance-based functional redundancy, finally the interdependency index. We will provide examples of maximal taxon-based functional redundancy (Fig. 1B), maximal abundance-based functional redundancy (Fig. 1C) and minimal interdependency (Fig. 1D) by varying the maximum secretion profile of the species, while keeping the number of species in the sample and their abundances fixed.

Exceptions include the case of maximal reference taxon-based functional redundancy, where all species in the reference must be included in the sample (Fig. 1B (bottom)) and abundance-based redundancy where $S_{5}$ must be excluded from the sample to achieve optimal abundance-based redundancy.

In the toy example, shown in Fig. 1A, the sample taxon-based functional redundancy results in $R_{Taxon}=-0.90$, while the reference taxon-based functional redundancy measure amounts to $R_{Taxon}=-1.24$. Maximal sample taxon-based functional redundancy occurs if every microbe within the sample exhibits an identical quantitative metabolic function, which manifests as a uniform distribution within the feature vector $\tilde{\boldsymbol{f}}$ of relative frequencies ($\tilde{\boldsymbol{f}}=\left( \frac{1}{5},\frac{1}{5},\frac{1}{5},\frac{1}{5},\frac{1}{5} \right)$ (Fig. 1B (top)). In comparison, for achieving maximal reference taxon-based functional redundancy, all seven species that can perform the function need to be in the sample and perform the same amount of maximal secretion ($\tilde{f}_{i}=\frac{1}{7}$ of ${\tilde{\boldsymbol{f}}}_{ref}$ for all $i\in\{1,2,3,4,6,7,8\}$) (Fig. 1B (bottom)), which would lead to $R_{Taxon}=0$ (reference-based). Note that we let $S_{5}$ in the sample and therefore sample taxon-based redundancy is not maximal (Figure 1B (bottom)).

In general, when at least one species cannot perform the function of interest, and the number of species that can perform the function is kept fixed, lower bounds of $R_{Taxon}$ can be calculated. For example, let $\tilde{\boldsymbol{f}}= (\tilde{f}_{1},\tilde{f}_{2},\ldots,\tilde{f}_{k},0,\ldots,0)$ be an $n$-sized vector, consisting of $0<k<n$ non-zero entries. Then$-D_{KL}\left( \tilde{\boldsymbol{f}}||\boldsymbol{U}_{n} \right)$ is maximal if and only if $\tilde{f}_{1}=\tilde{f}_{2}=\ldots=\tilde{f}_{k}=\frac{1}{k}$ and we can determine upper bounds (maximal functional redundancy) for both the sample and reference taxon-based functional redundancy measures, as they only differ in the number of zeros in the feature vector (Attribute 5). In our toy example, based on the information that one species in the sample cannot perform the function, the upper bound of the sample taxon-based functional redundancy measure is -0.22. Based on the information that, out of the total five species in the sample, only S₅ cannot perform the function ($k=4$), the upper bound (maximal functional redundancy) using the reference taxon-based measure would be -0.56 ($m=7$).

The taxon-based functional redundancy measures ignore the information given by the community composition. The difference between taxon-based functional redundancy and abundance-based functional redundancy becomes clear when comparing maximal sample taxon-based functional redundancy (Fig. 1B (top)) and maximal abundance-based functional redundancy (Fig. 1C). While in the scenario of maximal sample taxon-based redundancy, every species contributes equally to the total community output, the scenario of maximal abundance-based redundancy describes the situation where each species contributes in proportional to its abundance.

For calculating the interdependency index, the Kullback-Leibler divergence is calculated between all shares of non-zero community secretion $\left( \tilde{f}_{j} \right)_{j\in J}$, $j\in J=\{1,2,3,4\}$ ($\left( \tilde{f}_{j} \right)_{j\in J}=(0.8,0.1,0.05,0.05)$ and the normalised species abundance vector (${\tilde{\boldsymbol{a}}}_{J}=(0.5,0.25,0.125,0.125)$), resulting in an interdependency index of $I=0.19$. Fig. 1D illustrates the scenario of minimal interdependency for the normalised abundances in the toy example. To achieve minimal interdependency ($I=0)$ all elements of $\left( \tilde{f}_{j} \right)_{j\in J}$ must be equal to the corresponding value of the normalised abundance vector ${\tilde{\boldsymbol{a}}}_{J}$, meaning no species-species interactions exist.

# **Supplementary Figures**


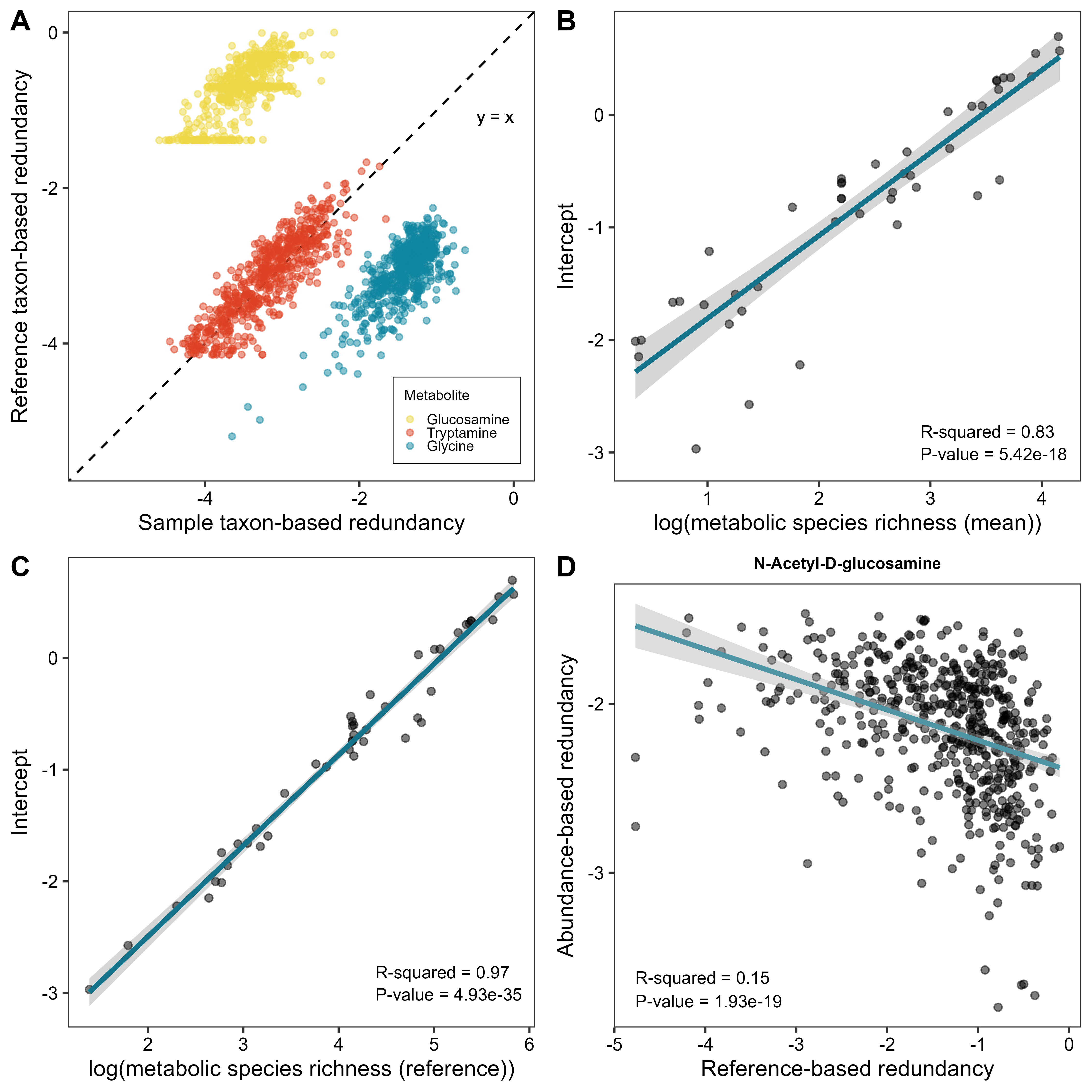


**Supplementary Figure 1:** Interrelations between measures of functional redundancy. A, Interrelations among glucosamine (yellow), tryptamine (red) and glycine (blue) between their taxon-based functional redundancy measures (dashed line: identity). Glucosamine has the fewest number of producing species in the reference (m = 4). For tryptamine, the reference contains m = 63 producing species, close to the average species richness in a modelled community (mean = 67.71, SD = 17.15). For glycine, the reference contains the highest number of producing species (m = 342). B, Scatterplot of the log-scaled average species richness of species that can secrete the metabolite (metabolic species richness (sample)) and the intercept of the regression model comparing the sample taxon-based with the reference taxon-based measure, with a fitted function (blue) and confidence band (grey). C, Scatterplot of the log-scaled number of species in the reference that can secrete the metabolite (metabolic species richness (reference)) and the intercept of the regression model comparing the sample taxon-based with the reference taxon-based measure, with a fitted function (blue) and confidence band (grey). D, Scatterplot for N-Acetyl-D-glucosamine, plotting the reference-based against the abundance-based measures, with the fitted regression line (blue) and confidence band (grey).


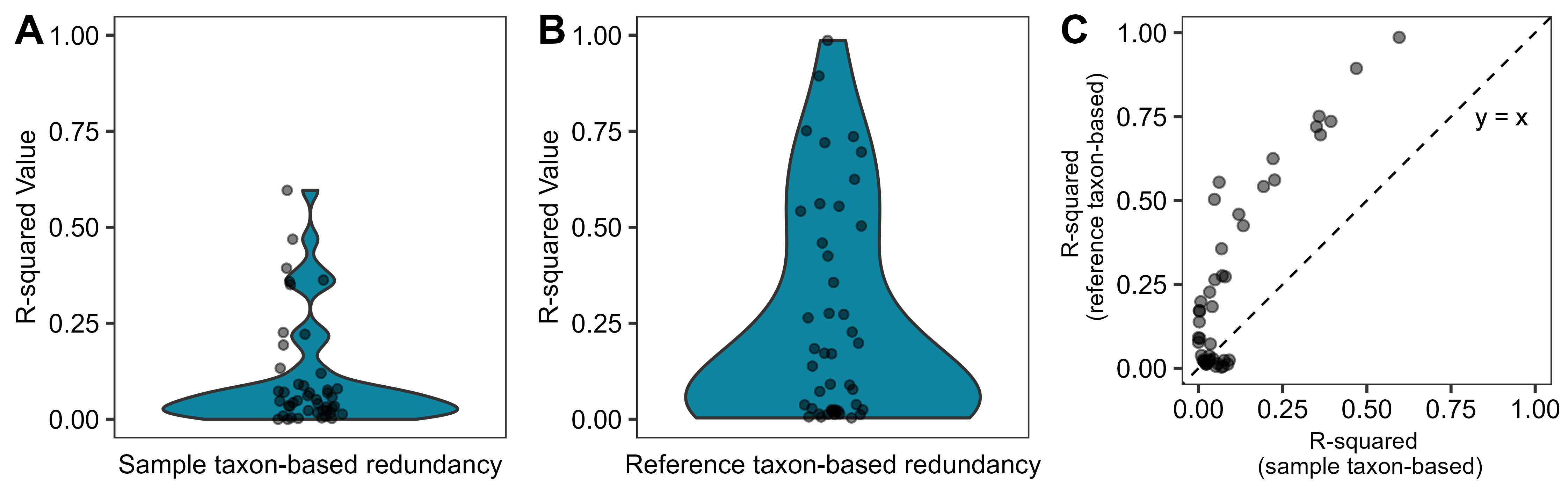


**Supplementary Figure 2:** Relationships between taxon-based functional redundancy measures and species diversity measured using Shannon entropy. A, Violin plot (blue) of R-squared values using sample taxon-based functional redundancy as the response variable. B, Violin plot (blue) of R-squared values using reference taxon-based functional redundancy as the response variable. C, Scatterplot showing R-squared values for each metabolite. The X-axis represents R-squared values from regression models treating the sample-based measure as response, while the Y-axis represents R-squared values from regression models using the reference-based measure as the response variable (dashed line: identity).


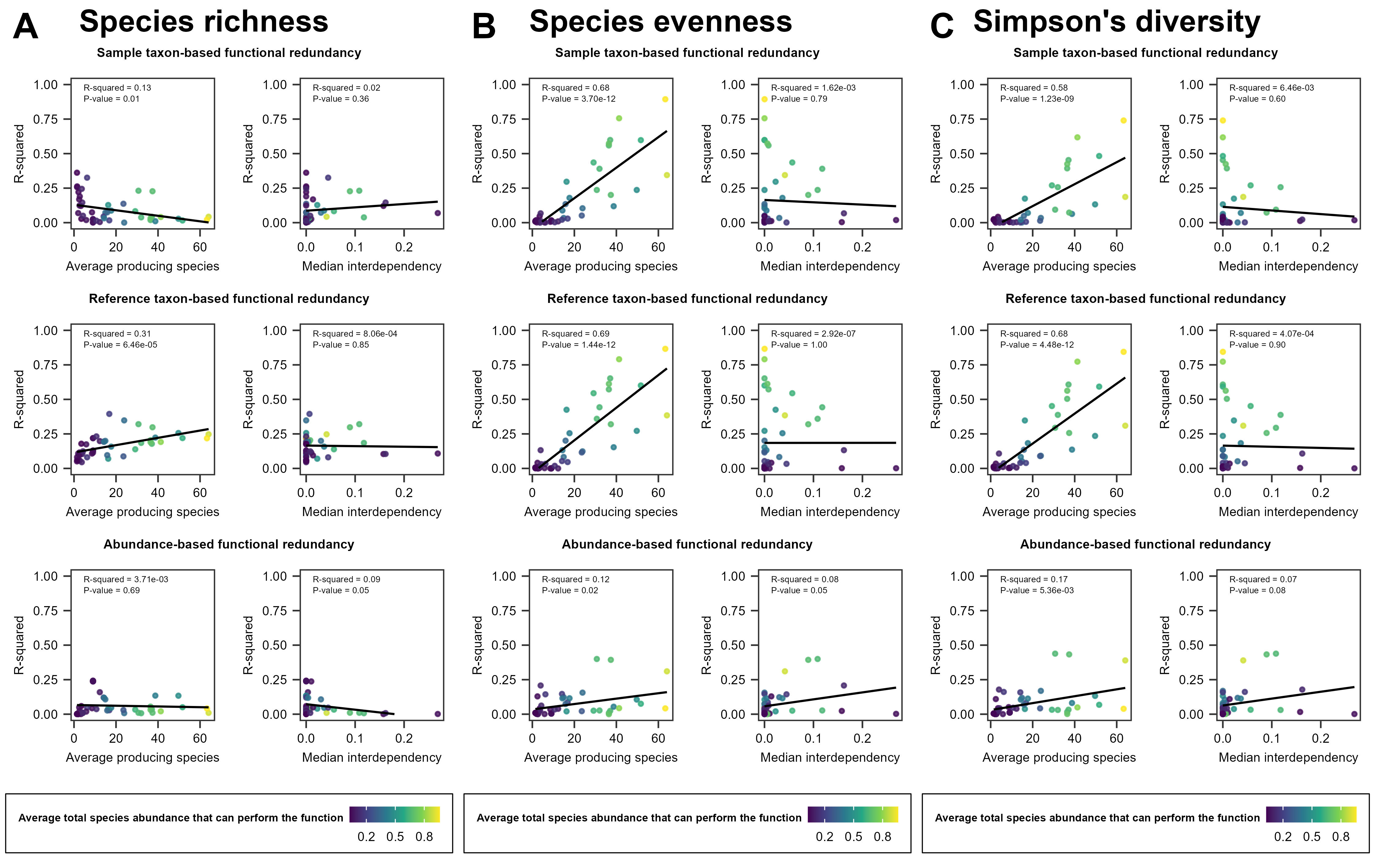


**Supplementary Figure 3:** Relationship between diversity metrics and functional redundancy of metabolites within the colorectal cancer (CRC) study. Scatterplots and regression lines for R-squared values between A, species richness, B, species evenness and C, Simpson’s diversity with measures of functional redundancy (Y-axis) plotted against the average number of producing species and the median functional interdependency index (X-axis). In each scatterplot, the metabolites are coloured based on their average total species abundance that can perform the function. The top row represents R-squared values (Y-axis, variance explained in sample taxon-based functional redundancy through species diversity) against average of species with the function in a sample and R-squared values (Y-axis, variance explained in sample taxon-based functional redundancy through species diversity) against median functional interdependency. The middle row represents R-Squared values (Y-axis, variance explained in reference taxon-based functional redundancy through species diversity) against average of species with the function in a sample and R-squared values (Y-axis, variance explained in reference taxon-based functional redundancy through species diversity) against median functional interdependency. The bottom row represents R-squared values (Y-axis, variance explained in abundance-based functional redundancy through species diversity) against average of species with the function in a sample and R-squared values (Y-axis, variance explained in abundance-based functional redundancy through species diversity) against median functional interdependency.
